# Ferritin Heavy Chain 1 Sustains Steroid Hormone Synthesis in Sheep Granulosa Cells by Suppressing Erastin-Induced Ferroptosis

**DOI:** 10.3390/biom16101507

**Published:** 2026-10-16

**Authors:** Ting Ge, Yifan Wen, Jiale Zhao, Wenjia Wang, Wenwen Xu, Bo Li, Yibing Wang, Weihua Zhang, Enping Zhang

**Affiliations:** Key Laboratory of Animal Genetics, Breeding and Reproduction of Shaanxi Province, College of Animal Science and Technology, Northwest A&F University, Yangling 712100, China; geting@nwafu.edu.cn (T.G.); wyf0162@126.com (Y.W.); jean000267@nwafu.edu.cn (J.Z.); wwjia@nwafu.edu.cn (W.W.); xww1227@nwafu.edu.cn (W.X.); lib1103@nwafu.edu.cn (B.L.); wangyibing@nwafu.edu.cn (Y.W.); zhangwh1211@nwafu.edu.cn (W.Z.)

**Keywords:** *FTH1*, ferroptosis, RNA-seq, granulosa cells, sheep

## Abstract

Litter size is a critical trait determining the economic efficiency of sheep production. Ferritin heavy chain 1 (*FTH1*), a key iron-storage protein, plays an important role in regulating cellular susceptibility to ferroptosis. Based on our previous observation that ferroptosis-related pathways differ between ovaries from single-lamb and multi-lamb Hu ewes, this study investigated the role of *FTH1* in regulating ferroptosis and granulosa cell (GC) function in sheep. *FTH1* expression was examined in ovarian tissues and GCs from Hu sheep with different reproductive phenotypes and follicular developmental stages using RT-qPCR. An erastin-induced ferroptosis model was established in sheep GCs, and *FTH1* function was evaluated through overexpression and interference experiments. Ferroptosis-related indicators, including ROS, C11-BODIPY 581/591, Fe^2+^, malondialdehyde (MDA), reduced glutathione (GSH), ferroptosis-associated genes, and GPX4 protein expression, were analyzed. Steroidogenic function was assessed by measuring the expression of *STAR*, *CYP11A1*, *HSD17B1*, and *3β-HSD*, as well as estradiol (E_2_) and progesterone (P_4_) secretion. RNA sequencing was performed to identify transcriptional changes associated with *FTH1* overexpression in erastin-treated GCs. *FTH1* expression was higher in ovaries from multiparous sheep and healthy follicles. *FTH1* overexpression alleviated erastin-induced ferroptosis by enhancing GPX4 expression and reducing Fe^2+^ accumulation, ROS production, and lipid peroxidation, whereas *FTH1* knockdown aggravated ferroptosis damage. Furthermore, *FTH1* maintained steroidogenic function by promoting *STAR* expression and preserving E_2_ and P_4_ synthesis under ferroptosis stress. Ferrostatin-1 (Fer-1) partially rescued GC viability and steroidogenic capacity, and attenuated erastin-induced apoptosis. Under erastin treatment, transcriptome analysis identified 297 differentially expressed mRNAs and 92 lncRNAs between *FTH1*-overexpressing GCs and the corresponding vector control. These differentially expressed transcripts represent candidate regulatory relationships that require further functional validation. Enrichment analysis highlighted cytokine signaling and ROS-related pathways, suggesting potential processes associated with the effects of *FTH1* under these conditions. Collectively, these findings support that *FTH1* protects sheep GCs against ferroptosis-induced injury and supports steroidogenic function, providing new insights into the role of iron homeostasis in follicular development.

## 1. Introduction

In meat sheep, follicular development directly determines ovulatory capacity and litter size, and is therefore a central determinant of reproductive performance [1]. Follicular atresia is a physiological process in the mammalian ovary that helps maintain ovarian microenvironmental homeostasis and eliminate follicles with poor developmental potential [2]. However, excessive follicular atresia reduces the pool of viable ovulatory follicles and can impair the reproductive performance of ewes [3].

Granulosa cells (GCs) are the major somatic cells that support follicular growth, oocyte maturation and endocrine function [4]. GC proliferation and differentiation are the key processes in follicular development [5]. They provide nutritional and paracrine support to oocytes through gap junctions, respond to gonadotropin stimulation, and synthesize steroid hormones such as estradiol and progesterone [1,5,6]. Ovarian steroidogenesis, which governs folliculogenesis and luteal development, relies on a synchronized enzymatic cascade to synthesize P_4_ and E_2_. Steroidogenic acute regulatory protein (*STAR*) initiates this pathway by mediating the rate-limiting transport of cholesterol across the outer-to-inner mitochondrial membrane [7]. Within the inner membrane, cytochrome P450 family 11 subfamily A member 1 (*CYP11A1*) cleaves cholesterol to yield pregnenolone [8]. Subsequently, 3β-hydroxysteroid dehydrogenase (*3β-HSD*) catalyzes the conversion of pregnenolone into P_4_, securing a vital hormone for luteal maintenance [9]. Downstream in the estrogenic pathway, 17β-hydroxysteroid dehydrogenase type 1 (*HSD17B1*) reduces low-potency estrone into bioactive E_2_ [10]. Together, the coordinated expression of *STAR*, *CYP11A1*, *3β-HSD*, and *HSD17B1* dictates systemic P_4_ and E_2_ dynamics in livestock reproduction.

Dysregulated GC death is a central event in follicular atresia. Apoptosis has long been regarded as the predominant mode of GC loss during this process [11,12,13]. More recent studies in mammalian and avian follicles have associated iron accumulation, impaired antioxidant defence and lipid peroxidation with GC dysfunction and follicular atresia [14,15,16,17,18]. However, the position of ferroptosis within the atretic process remains unresolved. Many ferroptosis-associated changes overlap with the broader oxidative deterioration of atretic follicles, making it difficult to determine whether ferroptosis initiates follicular degeneration, develops in parallel with apoptosis or occurs as a secondary consequence of cellular injury. The temporal and functional relationship between ferroptosis and apoptosis also remains unclear. These uncertainties are particularly relevant in sheep, for which direct evidence linking ferroptosis regulators to GC survival and endocrine function remains limited.

Ferroptosis is a novel regulated form of cell death driven by iron-dependent lipid peroxidation and characterized by labile iron accumulation, ROS production, membrane lipid peroxide overload, decline in cellular antioxidant capacity, and subsequent oxidative cell death [19]. Specifically, *ACSL4* enriches membranes with polyunsaturated fatty acids to facilitate lipid oxidation [20], while free iron catalyzes Fenton reactions to drive peroxidative cascades, yielding toxic MDA [21,22]. Conversely, *SLC7A11* imports cystine for glutathione (GSH) synthesis [19], enabling *GPX4* to reduce lipid peroxides into non-toxic alcohols [23]. Thus, the equilibrium between *ACSL4*/iron-driven MDA accumulation and the *SLC7A11*/GSH/*GPX4* antioxidant axis dictates cellular fate during ferroptosis. The core mechanism of ferroptosis relies on the production of harmful free radicals via the Fenton reaction, which is initiated by ferrous ions (Fe^2+^) [24]. Ferritin heavy chain 1 (*FTH1*) is a central component of ferritin and plays a critical role in iron storage and ferroptosis control [25]. By oxidizing Fe^2+^ and facilitating iron sequestration within ferritin, FTH1 stabilizes the labile iron pool and limits iron-catalyzed oxidative reactions [26]. In addition, ferritin turnover and *NCOA4*-mediated ferritinophagy govern iron release, thereby changing the cellular threshold for ferroptosis [27]. Suppression of ferritin degradation via this pathway ultimately inhibits ferroptosis [28]. These properties suggest that *FTH1* may act as a context-dependent regulator of ferroptosis, but its functional role needs to be elucidated in sheep ovarian cell types.

Our previous single-cell transcriptomic study identified differences in ferroptosis-related pathways between Hu sheep with different litter sizes and highlighted *FTH1* as a candidate gene associated with GC function [29]. However, whether altering *FTH1* expression changes the susceptibility of sheep GCs to ferroptosis-associated injury, and whether this response is accompanied by preservation of steroid hormone production, remained unknown. We hypothesized that *FTH1* limits erastin-induced iron and oxidative damage in sheep GCs and thereby helps preserve their viability and endocrine function. Erastin was selected to provide a defined ferroptotic challenge because it inhibits system xc^−^-mediated cystine uptake, impairs GSH-dependent antioxidant defence, and promotes lipid peroxidation [19]. To test this hypothesis, we combined *FTH1* overexpression and interference with erastin treatment and ferrostatin-1 rescue. Iron handling, antioxidant defence, lipid peroxidation, cellular viability, apoptosis, steroidogenic markers and E_2_ and P_4_ concentrations were assessed, while RNA sequencing was used to explore transcriptional changes associated with *FTH1* overexpression under erastin-induced stress. This design aimed to define the protective role of *FTH1* in the present sheep GC model and identify candidate molecular responses for further validation.

## 2. Materials and Methods

### 2.1. Ethical Approval and Consent to Participate

All animal-related procedures were performed in accordance with relevant guidelines and regulations. The study was approved by the Animal Care and Use Committee of Northwest A&F University, China (Approval No. DK2021113).

### 2.2. Collection of Ovaries and Follicles

Ovarian tissue samples from Hu sheep with different lambing numbers were obtained from our previous study [29]. Three ewes were used per group (n = 3 per group), with each ewe contributing one ovarian tissue sample for analysis. After collection, ovarian tissues were immediately immersed in RNA preservation solution, transported to the laboratory, and stored at −80 °C until RNA extraction. A total of 100 ovaries were collected from 50 non-pregnant ewes aged 1–2 years at a local slaughterhouse during winter. Their parity was unknown. Immediately after collection, ovaries were trimmed to remove excess connective tissue and transferred into pre-warmed sterile physiological saline (37 °C) supplemented with 100 IU/mL penicillin and 50 μg/mL streptomycin. Under a stereomicroscope, follicles were classified according to diameter as large follicles (>4.5 mm), medium follicles (3.0–4.5 mm), and small follicles (<3.0 mm). Medium follicles were further classified as healthy or atretic follicles based on morphological criteria. Specifically, healthy follicles appeared relatively translucent, with a pale pink or yellow surface and blood vessels evenly distributed across the theca folliculi. Atretic follicles were characterized by flocculent material inside, small dark patches on the follicular wall, a grayish-white appearance, and sparse blood vessels, accompanied by relatively turbid follicular fluid (Figure S1A). Granulosa cells from 8 to 12 follicles of the same category collected from multiple ewes were pooled to generate one sample. Each pooled sample was used as the unit of analysis; individual follicles, ovaries, and donor ewes within a pool were not counted as separate replicates. Each pooled sample was prepared from a separate, non-overlapping set of donor ewes and was therefore considered an independent biological replicate. Granulosa cell apoptosis is an important criterion for determining follicular atresia [30]. Therefore, we additionally examined the expression of apoptosis-related genes in atretic follicles (AF) and healthy follicles (HF), further supporting the accuracy of the follicle morphological classification (Figure S1B). These samples were used only for the detection of *FTH1* expression in subsequent experiments.

### 2.3. Isolation, Culture and Identification of GCs

Ovaries were collected from cyclic non-pregnant ewes at a slaughterhouse in Weinan, China. 100 ovaries from 50 ewes were obtained through multiple independent collections, placed in a thermos flask containing physiological saline maintained at 37 °C, and transported to the laboratory. GCs were isolated and cultured as previously described [31], with minor modifications. Briefly, ovaries were washed two to three times with pre-warmed sterile saline containing 1% penicillin–streptomycin and transferred to dishes containing mDPBS. Healthy follicles (2–6 mm in diameter) were punctured with a sterile syringe to collect follicular fluid, which was filtered through a 40 μm strainer and centrifuged at 1000 rpm for 5 min. ACK lysis buffer (R1013, Solarbio, Beijing, China) was then added to the pellet to remove red blood cells. The cells were subsequently washed twice with mDPBS, with each wash followed by centrifugation at 1000 rpm for 5 min. The cells were cultured in DMEM/F12 medium (C11330500BT, Gibco, Grand Island, NY, USA) supplemented with 10% FBS (F101-01, Nanjing Vazyme Biotech Co., Ltd., Nanjing, China) and 1% penicillin–streptomycin (BL505A, Biosharp, Hefei, China) at 37 °C in a humidified atmosphere containing 5% CO_2_. After 24 h of primary culture, the spent medium was removed to eliminate non-adherent cells and cellular debris, and fresh complete medium was added. The remaining adherent cells were maintained for subsequent experiments. Following 48 h of culture, cells were passaged at approximately 90% confluence using fivefold-diluted 0.25% trypsin-EDTA (5-fold, BL512A, Biosharp, Hefei, China) and seeded into appropriate culture plates for subsequent experiments. For GC identification, cells were seeded into 6-well plates and subjected to immunofluorescence staining at 70–80% confluence after 24 h of culture. The identity of GCs was confirmed by FSHR immunofluorescence staining (1:250, 22665-1-AP, Proteintech, Wuhan, China), as described previously [32] and in the Supplementary Materials (Figure S2). The collected granulosa cells were subsequently used for functional studies of *FTH1*.

### 2.4. Vector Construction, siRNA Synthesis and Transfection of GCs

The full-length coding sequence of sheep *FTH1* (NM_001009786.2) was shown in Table S1 and inserted between *Hind* III and *BamH* I cloning sites of the pcDNA3.1 vector to generate the overexpression plasmid oe-*FTH1*, and the empty pcDNA3.1 vector was used as the negative control. The plasmids were synthesized by Sangon Biotech (Shanghai, China). Subsequently, Sanger sequencing was performed on the *FTH1* overexpression plasmid, and sequence alignment verified successful plasmid construction. The *FTH1*-specific siRNA (si-*FTH1*) and the nontargeting negative control siRNA (NC) were also synthesized by Sangon Biotech (Table 1). Transfection was performed using Lipofectamine 3000 (Thermo Fisher Scientific, Waltham, MA, USA) according to the manufacturer’s protocol. In brief, both Lipofectamine 3000 and P3000 were used for plasmid transfection, while only Lipofectamine 3000 was applied for siRNA transfection without P3000. The culture medium was replaced at 4–6 h after transfection, and cells were collected at 24 h post-transfection. Furthermore, according to the results in Figure S4, transfection with 1.0 μL of Lipofectamine 3000 for 24 h was adopted as the final transfection protocol.

### 2.5. Preparation and Application of Erastin and Fer-1

A 10 mM erastin stock solution (E126853, Aladdin, Shanghai, China) was prepared by dissolving 5 mg of erastin in 914.01 μL dimethyl sulfoxide (DMSO, D8371, Solarbio, Beijing, China), as previously described [33]. Fer-1 is a classic highly selective ferroptosis inhibitor that blocks the lipid peroxidation chain reaction by scavenging lipid peroxyl radicals and is widely used in ferroptosis-related mechanistic studies [34]. A 10 mM Fer-1 (S7243, Selleck, Shanghai, China) stock solution was prepared by dissolving 1 mg of Fer-1 in 381.17 μL DMSO. Stock solutions were aliquoted and stored at −20 °C to avoid repeated freeze–thaw cycles. For cell treatment, stock solutions were diluted to the indicated working concentrations in complete culture medium. Erastin served two purposes in this study. First, it was used to establish a ferroptosis model in GCs. After treatment of the cells with the working concentration for 24 h, the erastin-containing culture medium was removed, and the cells were then transfected with oe-*FTH1* or si-*FTH1* together with their respective negative controls. The transfection operation is the same as that in Section 2.4. The role of *FTH1* in alleviating erastin-induced ferroptosis was subsequently investigated. Second, GCs were co-treated with erastin and Fer-1 for 24 h, and the samples were then collected to preliminarily investigate the relationship between ferroptosis and apoptosis in GCs. In the *FTH1* functional experiments, the final DMSO concentrations during exposure to 10 and 20 μM erastin were 0.1% and 0.2% (*v*/*v*), respectively. Within each experimental comparison, the treatment and corresponding control groups received the same final DMSO concentration. In the Fer-1 rescue experiment, the final DMSO concentrations were 0.2% and 0.3% (*v*/*v*) in the experiments using 10 and 20 μM erastin, respectively. At each erastin concentration, the erastin-only group received an equivalent amount of DMSO in place of the Fer-1 stock solution, ensuring the same final vehicle concentration within each comparison.

### 2.6. Experimental Assays and Data Collection

#### 2.6.1. Flow Cytometric Analysis of Apoptosis

Fer-1 was used at a final concentration of 10 μM. Cells were seeded in 6-well plates and treated when they reached 70–80% confluence. Then, GCs were treated with Fer-1 and erastin simultaneously for 24 h. This experiment had four treatment groups, namely E10 group (treated with 10 μM erastin only), E10 + Fer-1 group (treated with 10 μM erastin + 10 μM Fer-1), E20 group (treated with 20 μM erastin only), and E20 + Fer-1 group (treated with 20 μM erastin + 10 μM Fer-1). After 24 h of treatment, apoptosis was assessed using an Annexin V-FITC/PI apoptosis detection kit (C1062S, Beyotime, Shanghai, China). Briefly, 5 × 10^4^ to 1 × 10^5^ cells were harvested, centrifuged, and resuspended in binding buffer. Annexin V-FITC and propidium iodide were added sequentially, and samples were incubated in the dark at room temperature for 10–20 min. Before flow cytometric analysis, the cell suspension was filtered through a 200-mesh sieve to obtain a single-cell suspension. For all subsequent GC-based experiments, each biological replicate consisted of a pooled GC preparation isolated from multiple ovaries obtained from different ewes within the same collection batch. The three biological replicates were generated from three independent ovary collection and cell isolation batches conducted on separate occasions. Unless otherwise stated, all cell-based experiments were performed using three biological replicates (n = 3). Flow cytometric analysis data are presented as the mean ± SEM.

#### 2.6.2. RT-qPCR Analysis of Gene Expression

SLC7A11, GPX4, ACSL4 and NCOA4 were chosen as ferroptosis marker genes as they represent the complementary regulatory components of ferroptosis: glutathione antioxidant defense, lipid peroxidation, and ferritinophagy-mediated iron homeostasis [20,23,35,36]. These nodes collectively reflect the onset, progression, and termination of erastin-induced ferroptosis in cells.

Total RNA was extracted and reverse-transcribed into cDNA for subsequent RT-qPCR analysis. Gene-specific primers were designed using NCBI BLAST (https://www.ncbi.nlm.nih.gov/tools/primer-blast/index.cgi?LINK_LOC=BlastHome, accessed on 1 September 2026) or obtained from previously published studies, and primer sequences are listed in Table 2. Quantitative PCR was performed using the indicated reaction system and cycling conditions described in the Supplementary Materials (Tables S2 and S3). Relative expression levels of target genes were calculated using the 2^−ΔΔCt^ method within groups treated with the same concentration of erastin. The experiment was performed using three biological replicates (n = 3). Three technical replicates were performed for each biological replicate.

The specificity and amplification performance of 4 novel primer pairs (FTH1, ACSL4, STAR and HSD17B1) were validated before quantitative analysis. Amplification efficiencies were determined using standard curves generated from serially diluted cDNA and were within acceptable ranges (Table S4). Single peaks in melting curves indicated specific amplification, and product identities were further confirmed by Sanger sequencing. *β-actin* stability was evaluated by analyzing Cq values separately in three experimental series: *FTH1* overexpression or knockdown at 0, 10, and 20 μM erastin, and Fer-1 treatment at 10 and 20 μM erastin. Two-way ANOVA was performed, with residual normality and variance homogeneity assessed using the Shapiro–Wilk and Levene’s tests, respectively (all *p* > 0.05). No significant treatment-related changes in *β-actin* Cq values were detected (*p* > 0.05), supporting its use as the reference gene under the experimental conditions tested. Ct values > 35 were considered below the reliable detection limit.

#### 2.6.3. Western Blot Analysis

Western blotting was performed to examine proteins related to ferroptosis and steroidogenic function in GCs. Protein samples were separated by SDS-PAGE (PG223, EpiZyme, Shanghai, China) and transferred onto polyvinylidene difluoride membranes. Membranes were blocked with protein-free rapid blocking buffer (PS108P, EpiZyme, Shanghai, China) for 30 min at room temperature, incubated with primary antibodies overnight at 4 °C, and then incubated with HRP-conjugated secondary antibodies for 1.5 h at room temperature. Chemiluminescent signals were detected using ECL Detection Reagent (SQ203, EpiZyme, China) and visualized with a BIAS2800 imaging system (ENFAN, Xixian New Area, Shaanxi, China). Band intensity was quantified using ImageJ software (1.54g, National Institutes of Health, Bethesda, MD, USA) and, following background subtraction, target-protein band intensities were normalized to β-actin or GAPDH in the corresponding lanes and then expressed relative to the matched control group within each erastin concentration. This procedure was applied consistently across the relevant experiments. The following antibodies were used: GPX4 (Ab125066, Abcam, Cambridge, UK, 1:500), Caspase-3 (AB32351, Abcam, Cambridge, UK, 1:500), GAPDH (10494-1-AP, Proteintech, Wuhan, China, 1:20,000), β-actin (Proteintech, 66009-1-Ig, Wuhan, China, 1:50,000), CYP11A1 (YT1187, Immunoway, Suzhou, China, 1:750), STAR (12225-1-AP, Proteintech, Wuhan, China, 1:1000), FTH1 (Proteintech, 11682-1-AP, Wuhan, China, 1:750) HRP-conjugated Affinipure goat anti-mouse IgG (H + L) (RGAM001, Proteintech, Wuhan, China, 1:3000), and HRP-conjugated Affinipure goat anti-rabbit IgG (H + L) (SA00001-2, Proteintech, Wuhan, China, 1:300). The experiments were performed using three biological replicates (n = 3). Three technical replicates were performed for each biological replicate.

#### 2.6.4. Cell Viability Assay

Cell viability was measured using the Cell Counting Kit-8 (CCK-8; C0042, Beyotime, China). GCs were seeded into 96-well plates at a density of 1 × 10^4^ cells per well. After 24 h, the cells were treated with erastin alone or a combination of erastin and Fer-1 for an additional 24 h. Subsequently, 10 μL CCK-8 reagent was added to each well, and cells were incubated at 37 °C for an additional 3.5 h. Absorbance at 450 nm was then measured using a microplate reader. The experiment was performed using three biological replicates (n = 3).

#### 2.6.5. Measurement of Intracellular Reactive Oxygen Species (ROS) and Lipid Peroxidation

Intracellular ROS levels were assessed using the DCFH-DA fluorescent probe (CA1410, Solarbio, China). Cells were seeded in 96-well plates at a density of 1 × 10^4^ cells per well and treated as indicated. DCFH-DA was diluted 1:1000 in serum-free medium to a final concentration of 10 μM. After treatment, the culture medium was removed, 100 μL of diluted DCFH-DA was added to each well, and cells were incubated at 37 °C for 30 min. Cells were washed three times with serum-free medium to remove unincorporated probe. Green fluorescence was observed under an inverted fluorescence microscope with excitation and emission wavelengths of 488 and 510 nm, respectively. For each erastin concentration, the *FTH1*-overexpression or *FTH1*-interference group was compared with its corresponding control group. Fluorescence intensity was quantified using ImageJ. To avoid treating individual fields as independent biological replicates, the fluorescence intensity values from the three fields were averaged to obtain one mean value for each biological replicate, and these replicate-level means were used for statistical comparisons. The green fluorescence channel was extracted, and the background was subtracted using identical parameters for all images. Fluorescence-positive regions were selected using a fixed threshold, and measurements were obtained with Area, Mean gray value, Integrated density and “Limit to threshold” enabled. Positive objects were identified using fixed particle-size criteria. For each independently prepared pooled GC culture, which represented one biological replicate, integrated density measurements from multiple non-overlapping image fields were averaged to generate a single value. Therefore, three independent values per group, corresponding to the three biological replicates, were included in the statistical analysis. Pairwise comparisons were performed using an unpaired two-tailed Student’s *t*-test.

Lipid peroxidation was assessed using a C11-BODIPY 581/591 assay kit (S0043S, Beyotime, China). Cells were seeded in 96-well plates at a density of 1 × 10^4^ cells per well. After the indicated treatments, the culture medium was removed, and the cells were washed once with PBS. C11-BODIPY 581/591 was diluted to 2 μM in DF12 medium, and 100 μL was added to each well. Cells were incubated at 37 °C for 30 min, washed twice with PBS, and covered with PBS for fluorescence measurement using a microplate reader. Green fluorescence from the oxidized probe was measured at excitation/emission wavelengths of 488/510 nm, and red fluorescence from the reduced probe was measured at 581/591 nm. Lipid peroxidation was expressed as the ratio of green to red fluorescence. Lipid peroxidation was measured using a microplate reader in three independent experiments, with five technical replicate wells per group in each experiment. Technical replicates were averaged within each experiment before statistical analysis. Data are presented as the mean ± SEM (n = 3 independent experiments).

#### 2.6.6. Iron Assay

Total iron (Fe) and ferrous iron (Fe^2+^) levels were measured using commercial assay kits (catalog Nos. G1217W and G1212W; Geruisi-bio, Suzhou, China). This kit has a minimum detection limit of 0.15 µg/mL. Cells were collected from 60 mm dishes and centrifuged, and the supernatant was discarded. Approximately 2.5 × 10^6^ cells were resuspended in 1 mL extraction solution and sonicated on ice (200 W, 3 s on/10 s off, 30 cycles). After centrifugation at 12,000 rpm and 4 °C for 10 min, the supernatant was collected and kept on ice. Standards, blanks and samples were mixed with the kit reagents and incubated at room temperature for 15 min. Absorbance was measured at 562 nm, and total iron and Fe^2+^ contents were calculated according to the manufacturer’s instructions. The experiment was performed using three biological replicates (n = 3):

$${Total\;iron\;or\;{Fe}^{2+}}_{(\mu g/\mathrm{mL})}=\left(C_{standard}*V_{1}\right)*\frac{\left(A_{sample}-A_{blank}\right)}{\left(A_{standard}-A_{blank}\right)}/V_{1}*D$$

where A_sample_, A_blank_ and A_standard_ represent the absorbance values of the sample, blank, and iron/Fe^2+^ standard, respectively, C_standard_: concentration of iron/Fe^2+^ standard, 2 μg/mL; V1: volume of sample added, 0.06 mL; D: dilution factor.

#### 2.6.7. Glutathione Assay

Reduced glutathione (GSH) levels were measured using the Glutathione Assay Kit (A006-2-1; Nanjing Jiancheng, Nanjing, China). Cells were harvested from 60 mm dishes and lysed by sonication. A 0.2 mL aliquot of lysate was mixed with 0.2 mL Reagent I and centrifuged at 3500 rpm for 10 min. The supernatant was collected for protein concentration and GSH determination. Blanks, standards and samples were prepared according to the manufacturer’s instructions, and absorbance was measured at 405 nm using a microplate reader. GSH content was normalized to protein concentration. The relative contents of GSH were normalized to the blank control group, which was set as 100%. The experiment was performed using three biological replicates (n = 3):

$${GSH}_{(\mu \mathrm{mol}/gprot)}=\frac{{(A}_{sample}-A_{blank})}{\left(A_{standard}-A_{blank}\right)}*C_{standard}*2/Cpr$$

where A_sample_, A_blank_, and A_standard_ represent the absorbance values of the sample, blank, and GSH standard at 405 nm, respectively, C_standard_ is the concentration of the GSH standard substance, and Cpr is the protein concentration of the sample.

#### 2.6.8. Malondialdehyde (MDA) Quantification

MDA content was measured using the Malondialdehyde Assay Kit (A003-4-1, microplate method; Nanjing Jiancheng, China). Cells cultured in 60 mm dishes were collected into 1.5 mL centrifuge tubes and lysed with 0.3 mL Reagent V extraction solution. After ultrasonic disruption, protein concentration was determined by BCA assay. A 0.2 mL aliquot of each sample was used for MDA measurement according to the manufacturer’s protocol. Reaction mixtures were incubated in a water bath above 95 °C for 40 min, cooled under running water, and centrifuged at 4000 r/min for 10 min. Absorbance was measured at 530 nm using a microplate reader, and MDA content was normalized to protein concentration. The relative contents of MDA were normalized to the blank control group, which was set as 100%. The experiment was performed using three biological replicates (n = 3):

$${MDA}_{(\mathrm{nmol}/mgprot)}=\frac{A_{sample}-A_{blank}}{A_{standard}-A_{blank}}*C_{standard}/Cpr$$

where A_sample_ is the measured OD value, A_blank_ is the blank OD value, A_standard_ is the standard OD value, C_standard_ is the concentration of the standard substance, and Cpr is the protein concentration of the sample.

### 2.7. ELISA Detection of Estradiol and Progesterone Concentrations

GCs were seeded in 12-well plates at a density of 1.5 × 10^5^ cells per well and cultured for 24 h, by which time they had attached, spread and reached approximately 70–80% confluence. Corresponding treatments were then performed, and culture medium was collected 24 h thereafter. Cell-free wells containing the corresponding culture medium and treatments were incubated under the same conditions and used as background controls, and were also collected 24 h thereafter. Samples were centrifuged at 3000 rpm for 20 min at 4 °C, and the supernatant was collected for testing. Estradiol (E_2_) ELISA kits (F3907-A, FANKEW, Shanghai, China) and progesterone (P_4_) ELISA kits (F3911-A, FANKEW, China) were used to detect the hormone synthesis function of GCs. The detection range for E2 was 0.5–100 pg/mL, whereas that for P_4_ was 10–2000 pg/mL. The E_2_ limit of detection is <0.5 pg/mL, and intra-assay coefficients of variation <15%. The P_4_ limit of detection is <10 pg/mL, and intra-assay coefficients of variation <15%. A total of 50 μL of standard solutions, blank reagent, and 5-fold diluted cell supernatant were added to corresponding microwells, followed by incubation with HRP-conjugated specific antibodies at 37 °C for 60 min. After six washes, chromogenic substrates were added for 15 min of dark incubation. The reaction was terminated with stop solution, and the optical density (OD) at 450 nm was immediately measured via a microplate reader (Infinite F50, Tecan Group Ltd., Männedorf, Switzerland). Standard curves were established based on the OD values of serial standards to calculate the concentrations of E_2_ and P4 in samples. Hormone concentrations measured in cell-free controls were subtracted from those of the corresponding samples. Meanwhile, proteins were recovered from the TRIzol lysates of the corresponding culture wells [42] and quantified using BCA kit (PC0020, Solarbio, China), and the background-corrected hormone concentrations were normalized to the total cellular protein content of the corresponding wells. The experiment was performed using three biological replicates (n = 3), and results were presented as mean ± SEM.

### 2.8. Granulosa Cell Transcriptome Sequencing

To investigate transcriptomic changes associated with *FTH1* overexpression under ferroptosis stress, RNA sequencing was performed using GCs from the control group (10 μM erastin) and the treatment group (10 μM erastin + *FTH1* overexpression). Specifically, GCs were first treated with 10 μM erastin for 24 h, after which the erastin-containing medium was removed. Then, cells were transfected with empty vector or oe-*FTH1*. The medium was replaced 4-6 h after transfection, and cells were cultured until 24 h post-transfection. Total RNA was extracted using TRIzol reagent (15596018CN, Invitrogen, Carlsbad, CA, USA), and RNA integrity was assessed with an Agilent 5300 Bioanalyzer (RQN > 6.5). Libraries were constructed using the Illumina TruSeq RNA Library Prep Kit with Ribo-Zero Plus, and sequencing was performed on a NovaSeq X Plus platform by Majorbio Bio-Pharm Technology Co., Ltd. (Shanghai, China). Raw reads were quality-checked using FastQC and trimmed with Trimmomatic to remove adapters and low-quality reads. Clean reads were aligned to the reference genome (NCBI RefSeq Ensembl Ovis_aries_rambouillet.ARS-UI_Ramb_v2.0) using STAR. Gene expression was quantified using featureCounts, and differential expression analysis was performed with DESeq2 [43] using the criteria |log2 fold change| > 1.2 and *p* < 0.05. KEGG pathway enrichment analysis was conducted for differentially expressed genes, and *p* < 0.05 was considered significant. Transcripts were reconstructed using StringTie [44]. Known lncRNAs were identified by comparison with annotated lncRNAs in GFF/GTF files and lncRNA databases. Novel candidate lncRNAs were screened based on transcript length (>200 bp), exon number (≥2), and protein-coding potential predicted by CNCI [45] (score < 0), CPC [46] (score < 0.5), CPAT [47] (score < 0.5), and Pfam (E-value < 1 × 10^−3^ as the exclusion threshold). Transcripts predicted to have non-coding potential by these tools were retained as novel lncRNAs. The lncRNA identification pipeline—including the reference databases, initial transcript filtering criteria, coding-potential tools and thresholds, the intersection strategy, and the genomic classification of the identified lncRNAs—is described in detail in Supplementary Section S3. Construction of the candidate lncRNA–mRNA trans-regulatory network was shown in Supplementary Section S4. The raw sequencing data have been deposited in the Genome Sequence Archive (GSA) under accession number CRA033165. The complete information on sequencing quality, sequencing depth, mapping rate, percentage of reads assigned, and number of genes detected is shown in Tables S5–S7.

### 2.9. Statistical Analysis

Data are presented as the mean ± standard error of the mean (SEM). Statistical analyses were performed using SPSS 24.0 (IBM, Armonk, NY, USA). For the erastin concentration-screening CCK-8 assay, one-way ANOVA was used, followed by LSD multiple-comparison testing. For other experiments involving two-group comparisons under the same erastin concentration, unpaired two-tailed Student’s t-tests were used. In order to control the background effects of erastin exposure and transfection, statistical comparisons were restricted to matched groups within the same erastin concentration, and no comparisons were made among different erastin concentrations. Data were normalized to the corresponding control group under the same erastin concentration. *p* < 0.05 was considered statistically significant, and *p* < 0.01 was considered highly significant. Graphs and statistical annotations were generated using GraphPad Prism 8.0.2 (GraphPad Software, San Diego, CA, USA).

## 3. Results

### 3.1. Tissue Expression of FTH1 in Hu Sheep

**Figure 1:**
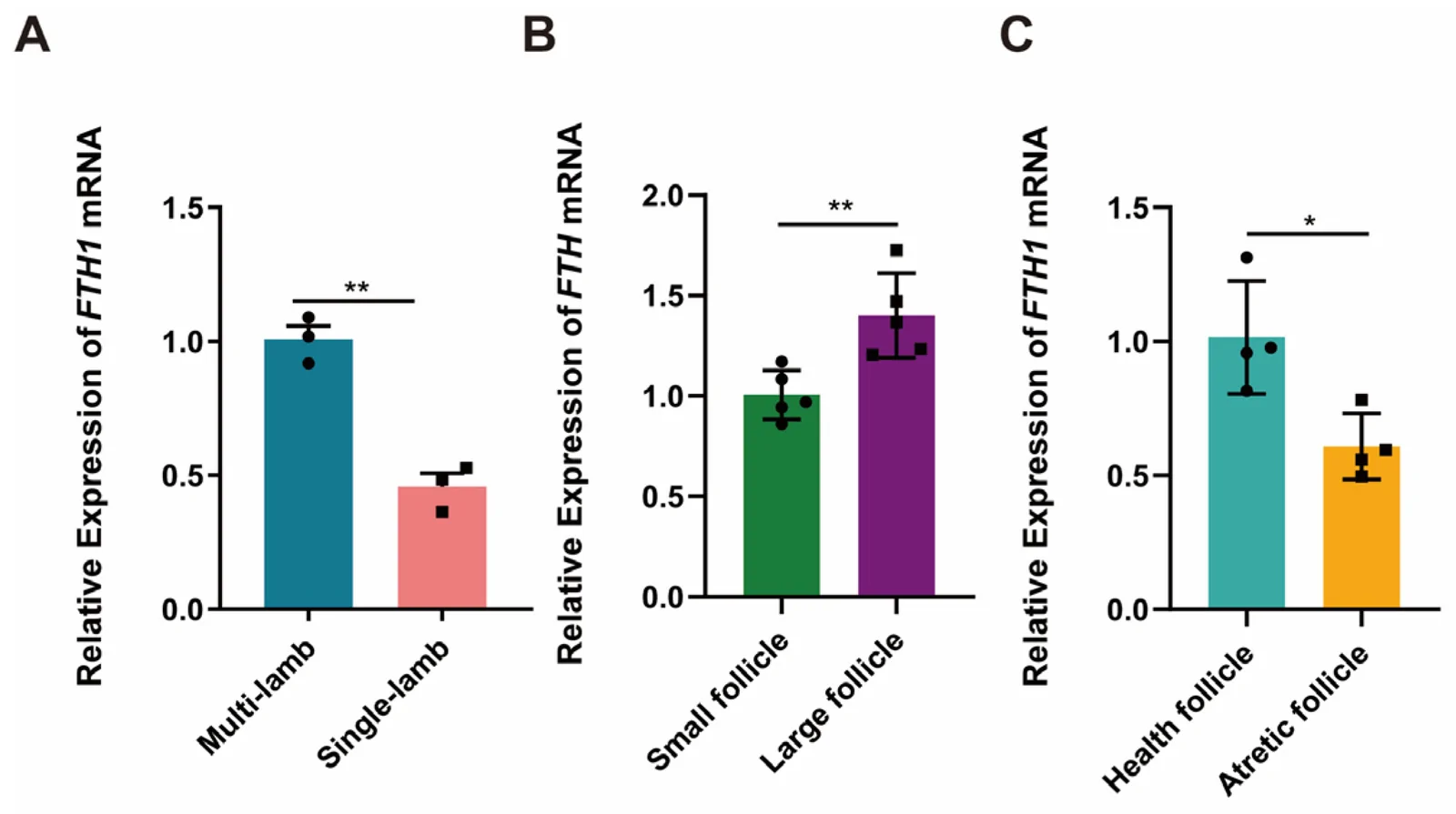
Expression of *FTH1* in Hu sheep ovarian tissues and granulosa cells. (**A**) Relative FTH1 mRNA expression in ovarian tissues from single-lamb and multiple-lamb Hu sheep. Each point represents one individual ewe (n = 3 animals per group). (**B**) Relative FTH1 mRNA expression in GCs from small follicles (<3.0 mm) and large follicles (>4.5 mm). Each point represents one independently prepared GC pool obtained by combining cells from 8 to 12 follicles (n = 5 pooled samples per group). (**C**) Relative FTH1 mRNA expression in GCs from healthy and atretic follicles measuring 3.0–4.5 mm in diameter. Each point represents one independently prepared GC pool obtained by combining cells from 8 to 12 follicles (n = 4 pooled samples per group). The pooled samples in (**B**,**C**) do not represent individual animals. Circles and squares indicate individual sampling units and are used to distinguish the groups. In panels (**A**–**C**), circles represent the multiple-lamb, small-follicle, and healthy-follicle groups, respectively, whereas squares represent the single-lamb, large-follicle, and atretic-follicle groups, respectively. Data are presented as the mean ± SEM, with individual sampling units shown. * *p* < 0.05; ** *p* < 0.01.

Based on our previous finding that *FTH1* was upregulated in ovaries from multiparous Hu sheep compared with those from single-lamb sheep, we first examined *FTH1* expression in ovarian tissues and GCs from follicles at different developmental states. *FTH1* expression was significantly higher in ovarian tissue from the multiple-lamb group than in that from the single-lamb group (*p* < 0.05; Figure 1A). In isolated GCs, *FTH1* expression was higher in large follicles (LF, >4.5 mm) than in small follicles (SF, <3.0 mm) (Figure 1B). In addition, *FTH1* expression was significantly higher in GCs from healthy follicles (HF) than in those from atretic follicles (AF) (Figure 1C). These results motivate further investigation into the biological function of *FTH1* in GCs.

### 3.2. Screening of Erastin Concentrations for Inducing Cellular Stress and Ferroptosis-Associated Injury in GCs

**Figure 2:**
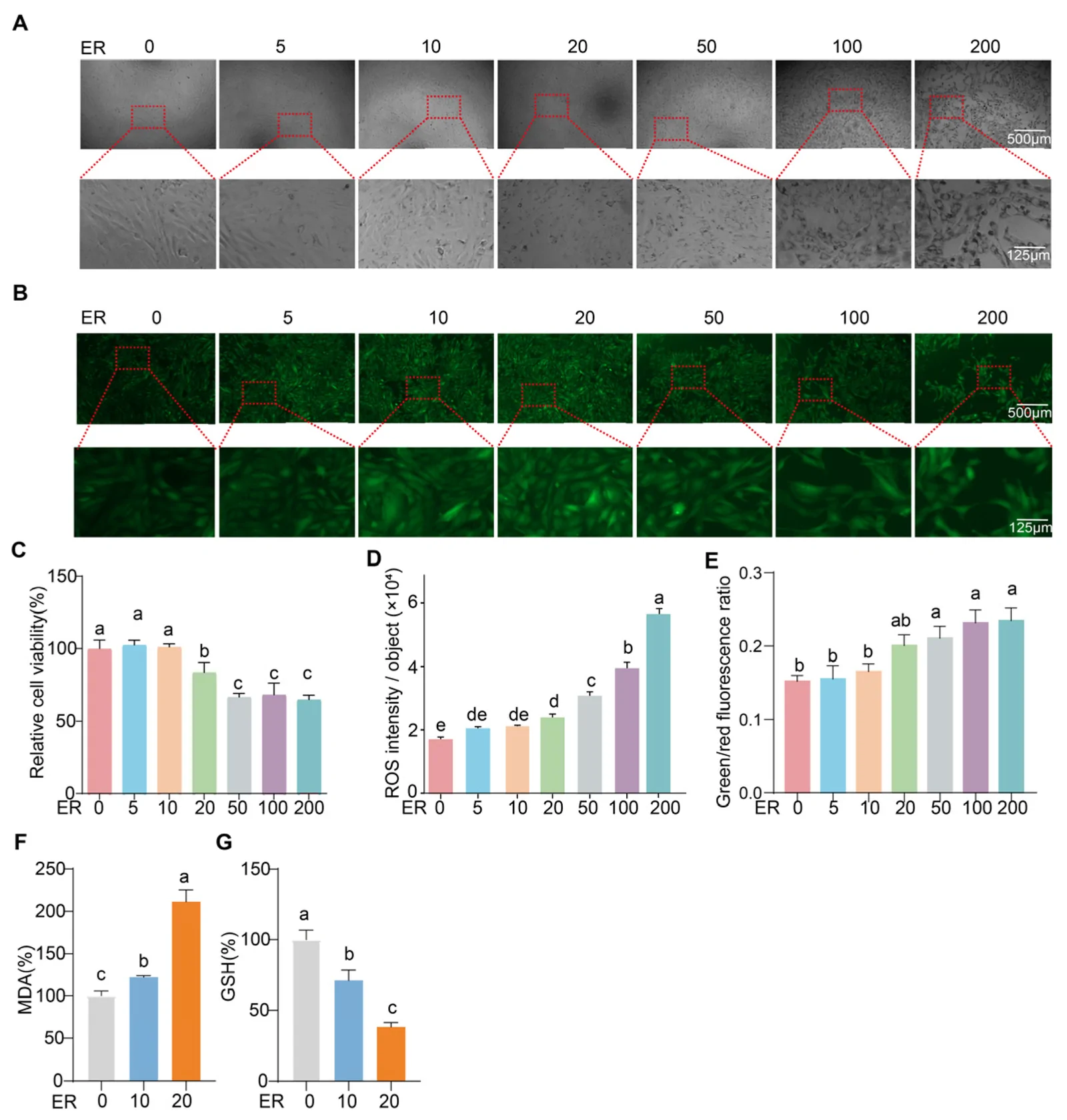
Dose-dependent effects of erastin on cellular stress and ferroptosis-associated injury in sheep granulosa cells. (**A**) Representative bright-field images showing erastin-induced morphological changes. The regions outlined by red dashed boxes are shown at higher magnification below. (**B**) Representative DCFH-DA fluorescence images showing intracellular ROS accumulation. (**C**) Relative cell viability measured using the CCK-8 assay. (**D**) Quantification of intracellular ROS fluorescence intensity. (**E**) BODIPY-based lipid peroxidation, expressed as the fold change in the green-to-red fluorescence ratio. (**F**) Relative MDA content following treatment with 0, 10 or 20 μM erastin. (**G**) Relative GSH content following treatment with 0, 10 or 20 μM erastin. Data are presented as the mean ± SEM from three independent biological replicates. Groups labelled with different lowercase letters differ significantly at *p* < 0.05. Scale bars: 500 μm and 125 μm.

Erastin is widely used to induce ferroptosis-associated oxidative injury. To identify suitable concentrations for subsequent functional analyses, sheep GCs were treated with 0, 5, 10, 20, 50, 100 or 200 μM erastin for 24 h. Cell morphology remained largely unchanged following treatment with 0–10 μM erastin. At 20 μM, erastin caused evident morphological abnormalities and reduced cell density, whereas more extensive cellular damage was observed at 50–200 μM (Figure 2A). ROS fluorescence intensity increased with erastin concentration. Compared with the untreated control, ROS accumulation was significantly increased at 20 μM and increased further at 50–200 μM (Figure 2B,D). The CCK-8 assay showed no significant reduction in cell viability at 5 or 10 μM. In contrast, cell viability was significantly decreased at 20 μM and was more markedly reduced at 50–200 μM (Figure 2C). Lipid peroxidation was further assessed using BODIPY 581/591 C11. The green-to-red fluorescence ratio showed an upward trend at 20 μM erastin and was significantly increased at 50–200 μM compared with the 0–10 μM groups (Figure 2E), indicating greater lipid oxidation at the higher erastin concentrations. Because treatment with 50–200 μM erastin caused severe cellular damage, these concentrations were not used in subsequent experiments. Further analysis of the selected concentrations showed that both 10 and 20 μM erastin significantly increased MDA accumulation and decreased intracellular GSH levels, with stronger changes observed at 20 μM (Figure 2F,G). Both concentrations also reduced E_2_ and P_4_ production (Figure S3). As shown in the results, erastin at 10 μM (a relatively low concentration) did not significantly reduce cell viability, corresponding to mild ferroptotic stress. By contrast, treatment with 20 μM (a higher concentration) caused a marked loss of cell viability, consistent with more severe oxidative injury. Accordingly, 10 μM erastin was selected to explore the *FTH1*-mediated regulatory mechanism under mild ferroptosis stress, while 20 μM was used to assess its influence under more severe oxidative injury.

### 3.3. Effects of FTH1 on Ferroptosis in GCs

**Figure 3:**
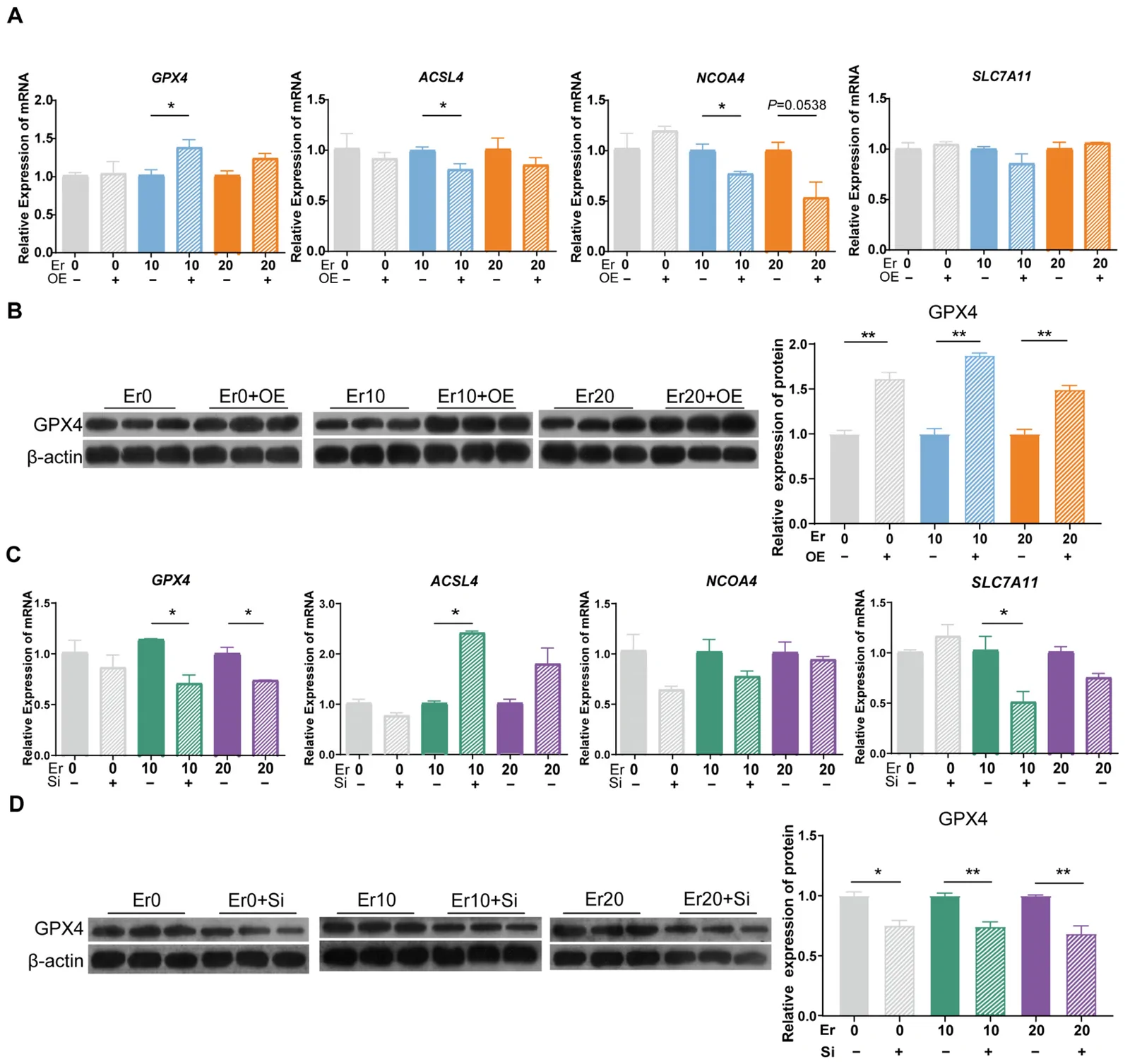
*FTH1* manipulation alters ferroptosis-related gene and protein expression in sheep granulosa cells. (**A**) Relative mRNA expression of *GPX4*, *ACSL4*, *NCOA4* and *SLC7A11* after *FTH1* overexpression. (**B**) Representative GPX4 Western blots and densitometric quantification after overexpression. (**C**) Relative mRNA expression after *FTH1* interference. (**D**) Representative GPX4 Western blots and densitometric quantification after interference. GCs were treated with 0, 10, or 20 μM erastin for 24 h. The erastin-containing medium was then removed, and cells were transfected with an empty vector or oe-*FTH1*, or with negative control siRNA or si-*FTH1*. The medium was replaced 4–6 h after transfection, and cells were cultured until 24 h post-transfection. Target-protein band intensities were normalized to β-actin in the corresponding lanes and then expressed relative to the matched control group within each erastin concentration. Data are mean ± SEM from three independent biological replicates. Comparisons were made between the matched *FTH1*-manipulated and control groups within each erastin concentration using Student’s *t*-test. * *p* < 0.05; ** *p* < 0.01.

*FTH1* overexpression and interference were confirmed at the mRNA and protein levels (Figures S5 and S6). Subsequently, we investigated whether *FTH1* regulates erastin-induced ferroptosis in GCs. Under 10 μM erastin treatment, *FTH1* overexpression significantly upregulated *GPX4* expression (*p* < 0.05) and downregulated *ACSL4* and *NCOA4* expression (*p* < 0.05). Under 20 μM erastin treatment, no significant differences were detected in the relative mRNA expression of *GPX4*, *ACSL4* or *NCOA4*, although *GPX4* showed an upward trend and *ACSL4* and *NCOA4* showed downward trends (Figure 3A). At the protein level, *FTH1* overexpression significantly increased GPX4 protein level with both 10 μM and 20 μM erastin treatment (*p* < 0.01; Figure 3B). In addition, under 10 μM erastin treatment, *FTH1* interference significantly downregulated *GPX4* and *SLC7A11* relative mRNA expression (*p* < 0.05) and upregulated *ACSL4* expression (*p* < 0.05; Figure 3C). *NCOA4* and *SLC7A11* were not significantly altered under this condition, although both showed downward trends. Under 20 μM erastin treatment, *FTH1* interference significantly reduced *GPX4* expression (*p* < 0.05), while *NCOA4* and *SLC7A11* showed downward trends (Figure 3C). Consistently, *FTH1* interference significantly reduced GPX4 protein level following treatment with either 10 or 20 μM erastin (*p* < 0.01; Figure 3D).

**Figure 4:**
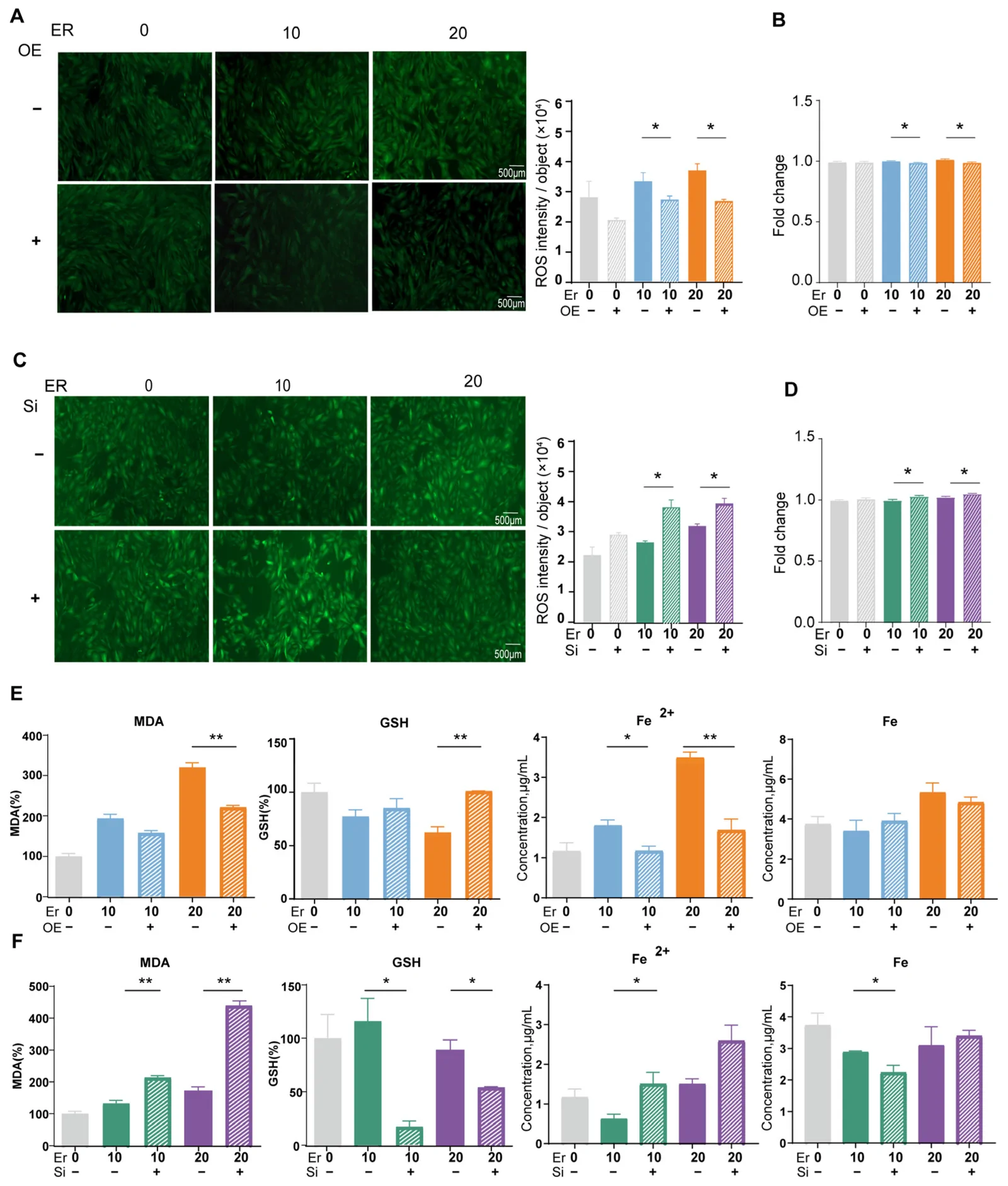
Effects of *FTH1* on erastin-induced ferroptosis in GCs. (**A**) Representative ROS fluorescence images and quantification after *FTH1* overexpression. (**B**) Lipid peroxidation levels in GCs after *FTH1* overexpression were assessed using C11-BODIPY 581/591. Lipid peroxidation was measured using a microplate reader, with five technical replicate wells per group in each experiment. (**C**) Representative ROS fluorescence images and quantification after *FTH1* interference. (**D**) Lipid peroxidation levels in GCs after *FTH1* interference were assessed using C11-BODIPY 581/591. Lipid peroxidation was measured using a microplate reader, with five technical replicate wells per group in each experiment. (**E**) Relative MDA and GSH and the concentrations of Fe^2+^ and total iron after overexpression. (**F**) The corresponding biochemical measurements after interference. GCs were treated and transfected as described in Figure 3. Target-protein band intensities were normalized to β-actin in the corresponding lanes and then expressed relative to the matched control group within each erastin concentration. Data are mean ± SEM from three independent biological replicates. Matched groups were compared within each erastin concentration using Student’s *t*-test. * *p* < 0.05; ** *p* < 0.01. Scale bar, 500 μm.

Ferroptosis-related biochemical indicators were also examined. *FTH1* overexpression significantly reduced ROS intensity at 10 and 20 μM (*p* < 0.05), whereas *FTH1* markedly increased it (*p* < 0.05, Figure 4A,C). Under 20 μM erastin treatment, *FTH1* overexpression significantly decreased MDA content (*p* < 0.01) and increased GSH levels (*p* < 0.01, Figure 4E). Iron assays further showed that *FTH1* overexpression significantly reduced intracellular Fe^2+^ accumulation (*p* < 0.05, Figure 4E). These biochemical changes indicate that *FTH1* overexpression attenuated ferroptosis-associated oxidative and iron-related injury in erastin-treated GCs. *FTH1* interference further aggravated erastin-induced ferroptosis damage, as indicated by significantly elevated MDA content (*p* < 0.01) and reduced GSH levels (*p* < 0.05) at concentrations of 10 μM and 20 μM. Meanwhile, si-*FTH1* increased intracellular Fe^2+^ accumulation while decreasing intracellular iron under the 10 μM erastin treatment (*p* < 0.05, Figure 4F). Together, these findings indicate that *FTH1* depletion aggravated ferroptosis-associated biochemical alterations following erastin exposure. To determine the effects of *FTH1*’s role in cellular iron, oxidative damage and antioxidant capacity, we further examined the effects of *FTH1* manipulation in the absence of erastin. In the absence of erastin, *FTH1* overexpression significantly increased GSH levels and decreased MDA, total iron and Fe^2+^ concentrations (*p* < 0.05; Figure S7A–D). Conversely, *FTH1* interference significantly increased MDA and Fe^2+^ concentrations. GSH levels were not significantly altered by *FTH1* interference in the absence of erastin, although a downward trend was observed (Figure S7A–D).

### 3.4. Effects of FTH1 on Steroidogenic Function in GCs

**Figure 5:**
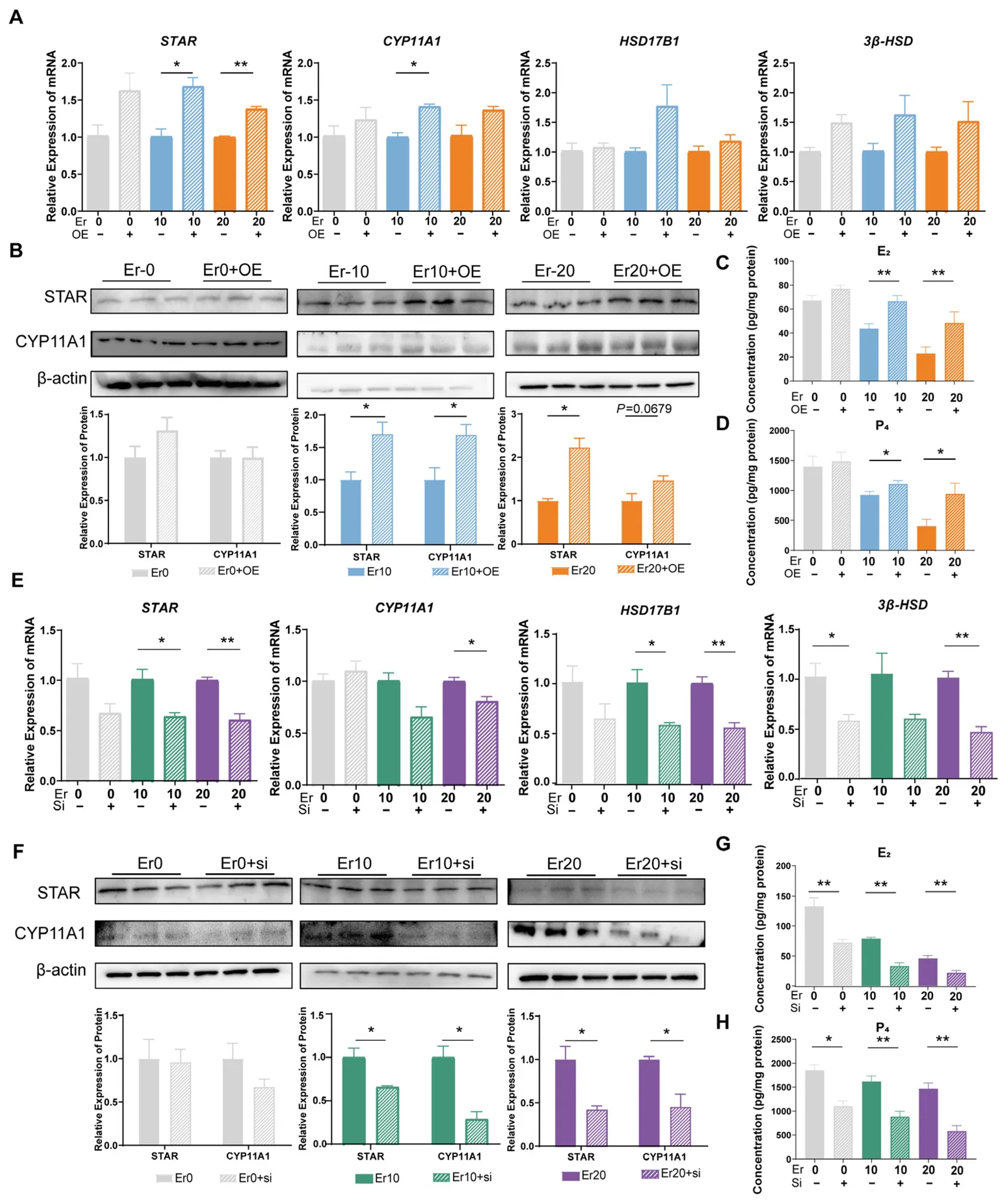
*FTH1* preserves steroidogenic markers and hormone production during erastin exposure. (**A**) RT-qPCR used to examine the relative expression of genes related to GC steroidogenic function after *FTH1* overexpression. (**B**) Western blotting used to examine the relative expression of proteins related to GC steroidogenic function after *FTH1* overexpression. (**C**) E_2_ and (**D**) P_4_ concentrations after overexpression. (**E**) Relative steroidogenic gene expression after *FTH1* interference. (**F**) Representative STAR and CYP11A1 Western blots and densitometric quantification after interference. (**G**): E_2_ and (**H**) P_4_ concentrations after interference. GCs were treated and transfected as described in Figure 3. Data are mean ± SEM from three independent biological replicates. Matched groups were compared within each erastin concentration using Student’s *t*-test. * *p* < 0.05; ** *p* < 0.01.

Because GCs are essential for follicular steroidogenesis, we next examined whether *FTH1* overexpression or knockdown affects hormone-related functional markers. Firstly, without erastin treatment, *FTH1* overexpression had no significant influence on the expression of hormone synthesis-associated genes (Figure 5A,B), nor did it alter E_2_ and P_4_ levels (Figure 5C,D). Knockdown of *FTH1* significantly decreased the mRNA level of *3β-HSD* (Figure 5E) and reduced E_2_ and P_4_ synthesis (Figure 5G,H). Under 10 μM erastin treatment, *FTH1* overexpression significantly upregulated *STAR* and *CYP11A1* expression (*p* < 0.05) and increased the expression trends of *HSD17B1* and *3β-HSD* (Figure 5A). Under 20 μM erastin treatment, *FTH1* overexpression markedly increased STAR expression (*p* < 0.01), while *CYP11A1*, *HSD17B1* and *3β-HSD* showed upward trends (Figure 5A). At the protein level, *FTH1* overexpression under 10 μM erastin significantly increased STAR and CYP11A1 levels (*p* < 0.05). Under 20 μM erastin, *FTH1* overexpression significantly increased STAR protein level (*p* < 0.05) and tended to increase CYP11A1 level (Figure 5B). In addition, under 10 and 20 μM erastin treatment, E_2_ and P_4_ concentrations were significantly higher in the *FTH1*-overexpression groups than in the corresponding erastin-treated control groups (*p* < 0.05, Figure 5C,D). *FTH1* interference also impaired steroidogenic function. Under 10 μM erastin treatment, *FTH1* interference significantly decreased *STAR* and *HSD17B1* relative mRNA expression (*p* < 0.05; Figure 5E). Under 20 μM erastin treatment, *STAR, CYP11A1, HSD17B1* and *3β-HSD* expression were suppressed after interference (*p* < 0.05; Figure 5E). At the protein level, *FTH1* interference significantly reduced STAR and CYP11A1 expression under both 10 μM and 20 μM erastin treatments (*p* < 0.05, Figure 5F). Under erastin treatment, *FTH1* interference significantly reduced E_2_ and P_4_ concentrations (*p* < 0.01; Figure 5G,H). Therefore, *FTH1* overexpression with erastin treatment partially improved steroidogenic function in sheep GCs, whereas *FTH1* knockdown further impaired it.

### 3.5. Fer-1 Partially Alleviates Erastin-Induced Functional Impairment and Apoptosis-Associated Changes in GCs

**Figure 6:**
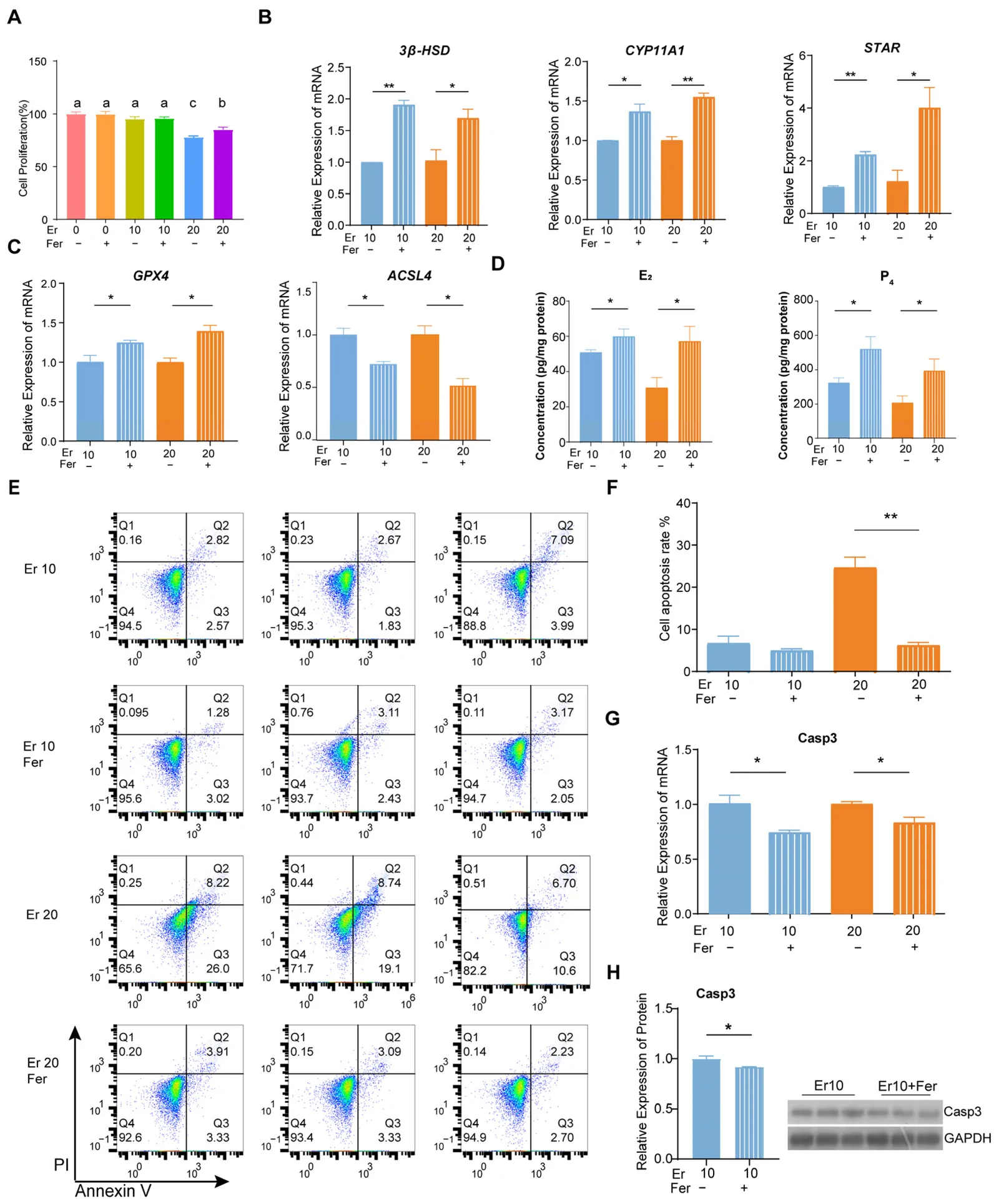
Effects of Fer-1 on erastin-induced functional impairment and apoptosis-associated changes in granulosa cells. (**A**) Cell viability detected by CCK-8. (**B**) Relative *3β-HSD, CYP11A1* and *STAR* mRNA expression detected by RT-qPCR. (**C**) Relative *GPX4* and *ACSL4* mRNA expression detected by RT-qPCR. (**D**) E_2_ and P_4_ concentrations detected by ELISA. (**E**) Representative Annexin V/PI flow-cytometry plots. (**F**) Quantified apoptotic-cell percentage. (**G**) Relative *Casp3* mRNA expression. (**H**) Representative caspase-3 Western blot and densitometric quantification. GCs were treated with 10 or 20 μM erastin with or without 10 μM Fer-1 for 24 h. Target-protein band intensities were normalized to GAPDH in the corresponding lanes and then expressed relative to the matched control group within each erastin concentration. Data are mean ± SEM from three independent biological replicates. Matched groups were compared within each erastin concentration using Student’s *t*-test. * *p* < 0.05; ** *p* < 0.01. Groups with different lowercase letters differ at *p* < 0.05.

To further clarify the relationship between ferroptosis and GC function, cells were co-treated with erastin and the ferroptosis inhibitor Fer-1. As shown in Figure 6A, 10 μM Fer-1 had no significant effect on cell viability, confirming that Fer-1 exerted no cytotoxicity against granulosa cells at this concentration. Meanwhile, subsequent molecular, steroidogenic, and apoptosis-related analyses focused on the effects of Fer-1 under erastin treatment. Therefore, comparisons were made between erastin-treated cells with and without Fer-1 co-treatment.

According to the CCK-8 assay, compared with 20 μM erastin alone, co-treatment with Fer-1 significantly restored cell viability (*p* < 0.05), indicating effective inhibition of ferroptosis-associated injury (Figure 6A). The results showed that Fer-1 co-treatment also upregulated *GPX4* and downregulated *ACSL4* expression (*p* < 0.05, Figure 6C). In addition, Fer-1 increased the expression of the steroidogenic markers *CYP11A1*, *STAR* and *3β-HSD* compared with erastin treatment alone (*p* < 0.05, Figure 6B). The ELISA results indicated that the E_2_ and P_4_ contents in the group treated with both Fer-1 and erastin were significantly increased (*p* < 0.05, Figure 6D).

Because GC apoptosis is closely associated with follicular atresia, we next examined whether apoptosis-related changes accompanied ferroptosis-associated injury during erastin exposure. Under 10 μM erastin treatment, Fer-1 co-treatment produced a non-significant downward trend in the apoptotic-cell percentage. Under 20 μM erastin treatment, Fer-1 significantly reduced the apoptotic-cell percentage (Figure 6E,F). Fer-1 also significantly decreased *CASP3* relative mRNA expression under erastin treatment (*p* < 0.05; Figure 6G), accompanied by lower Caspase-3 protein abundance (Figure 6H). Thus, the reduction in ferroptosis-associated injury following Fer-1 treatment coincided with lower apoptosis-related indices. These coordinated responses support an association or crosstalk between ferroptosis and apoptosis but do not establish that ferroptosis directly precedes or causes apoptosis in sheep GCs.

### 3.6. Exploratory RNA-Seq Analysis Identifies FTH1-Associated Transcriptional Changes Under Erastin-Induced Stress

**Figure 7:**
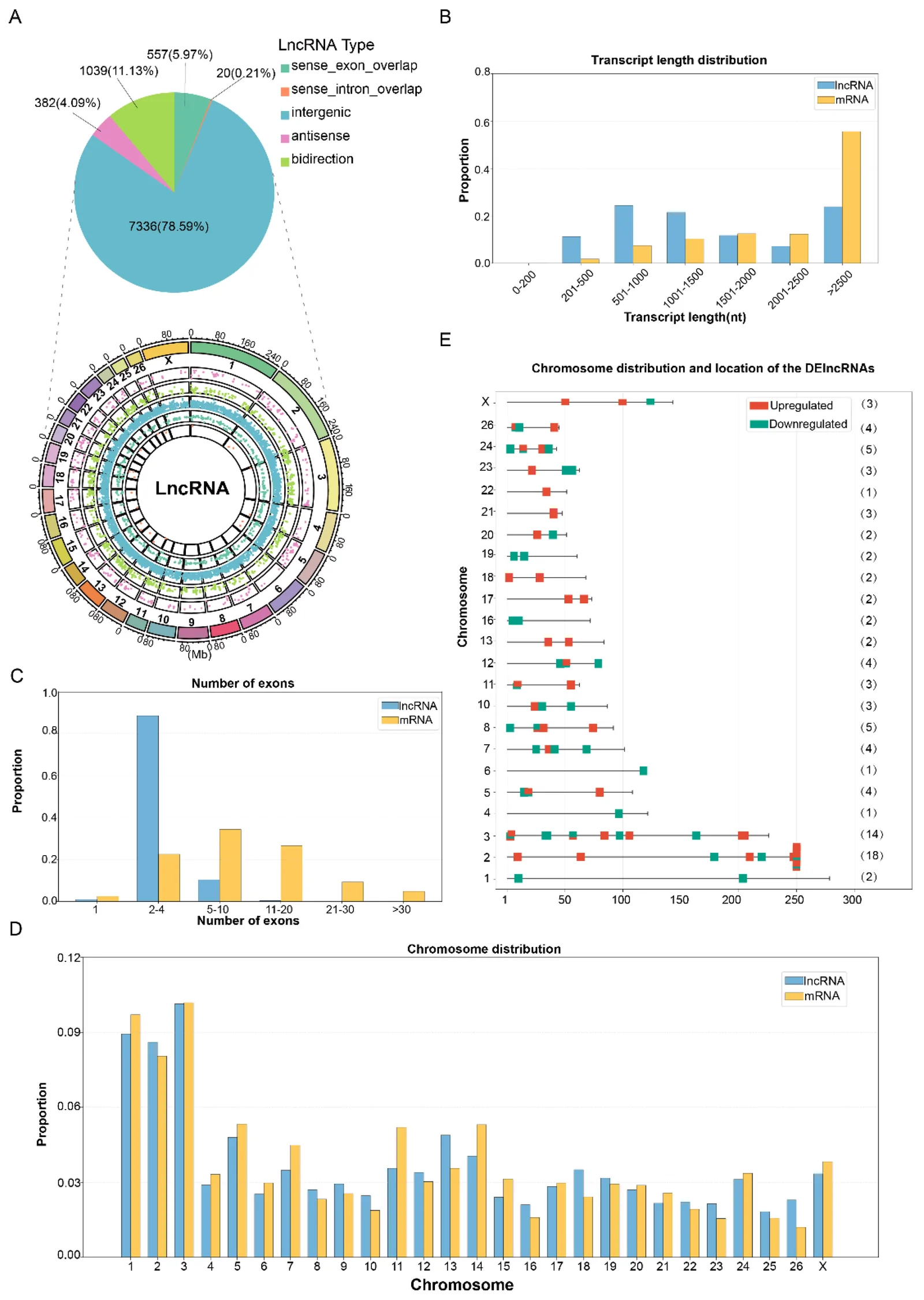
Genomic characteristics and structural features of identified lncRNAs. (**A**) Classification of lncRNAs based on genomic location (top) and a Circos plot showing their genome-wide distribution density (bottom). (**B**) Comparison of transcript length distribution between lncRNAs and mRNAs. (**C**) Comparison of exon number distribution between lncRNAs and mRNAs. (**D**) Chromosomal distribution of lncRNAs compared to mRNAs across all chromosomes. (**E**) Chromosomal locations and distribution of differentially expressed lncRNAs (DElncRNAs), with upregulated genes in red and downregulated genes in green. The numbers in parentheses indicate the count of DElncRNAs on each chromosome.

To explore potential mechanisms by which *FTH1* protects GCs under ferroptosis stress, transcriptome sequencing was performed on GCs treated with 10 μM erastin with or without *FTH1* overexpression. The sequencing data identified 18,988 annotated genes, 58,817 mRNA transcripts and 9334 lncRNA transcripts. To characterize the identified lncRNAs, we analyzed their genomic classification, structural features and chromosomal distribution. Based on genomic location relative to protein-coding genes, lncRNAs were classified as intergenic, bidirectional, sense exon-overlapping, sense intron-overlapping, or antisense lncRNAs. Intergenic lncRNAs accounted for the largest proportion (78.59%, 7336 transcripts), followed by bidirectional lncRNAs (11.13%, 1039 transcripts) and sense exon-overlapping lncRNAs (5.97%, 557 transcripts) (Figure 7A). A Circos plot further showed the genome-wide density and distribution of lncRNAs across chromosomes 1–26 and the X chromosome (Figure 7A). Compared with mRNAs, lncRNAs were generally shorter, with many lncRNAs ranging from 201 to 1000 nucleotides, whereas most mRNAs were longer than 2500 nucleotides (Figure 7B). LncRNAs also contained fewer exons, with approximately 90% containing two to four exons, whereas mRNAs showed a broader exon-number distribution (Figure 7C). LncRNAs and mRNAs showed broadly similar chromosomal distribution patterns, with the highest abundance on chromosomes 1, 2 and 3 (Figure 7D). Differentially expressed lncRNAs (DElncRNAs) were distributed across the genome, with chromosome 2 containing the highest number (18 transcripts), followed by chromosome 3 (14 transcripts) (Figure 7E).

**Figure 8:**
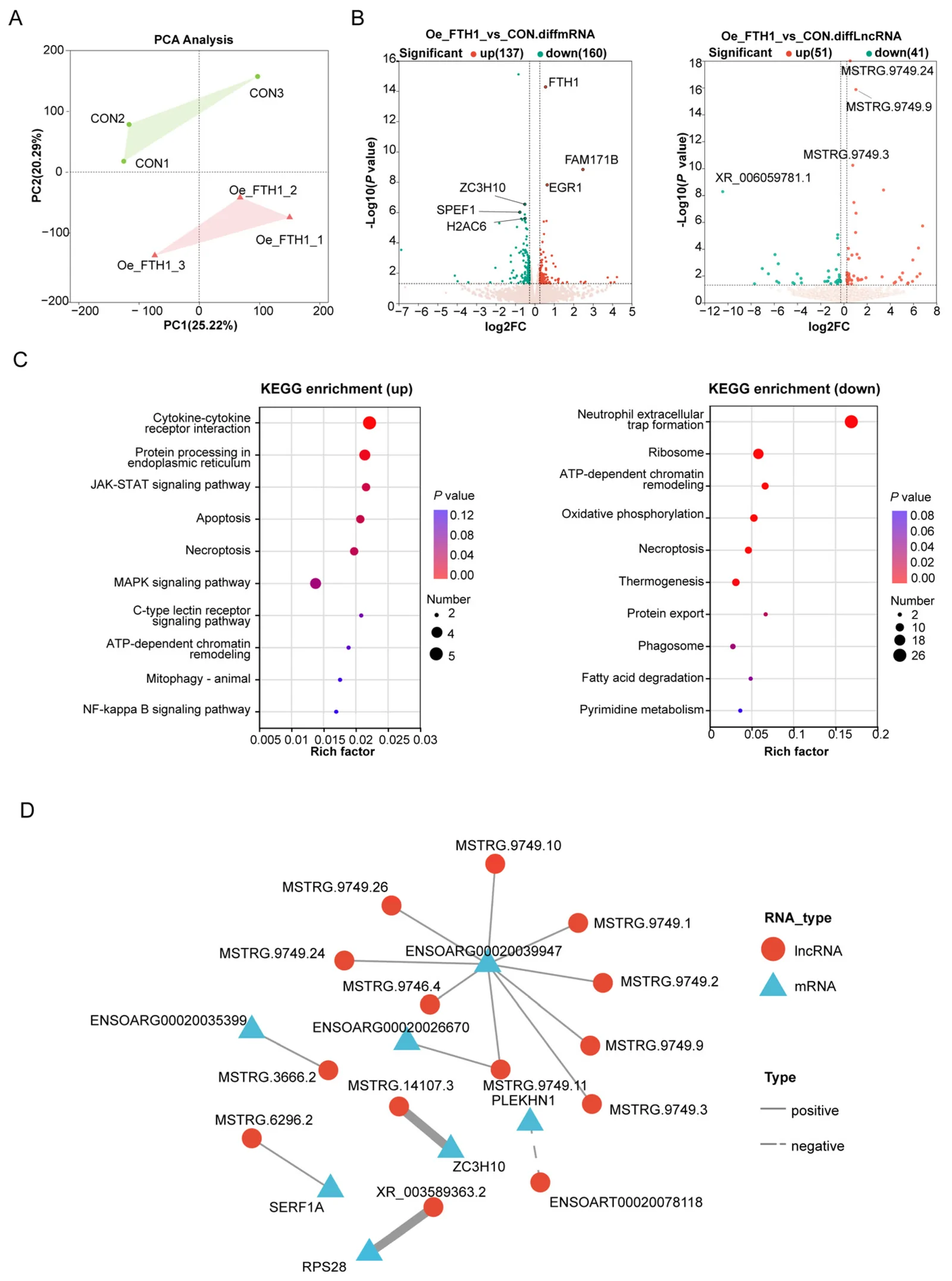
Transcriptional landscape and functional pathway enrichment in *FTH1*-overexpressing GCs under erastin-induced stress. (**A**) Principal component analysis (PCA) of transcriptomic profiles between the oe-*FTH1* and control groups. (**B**) Volcano plots of differentially expressed mRNAs (**left**) and lncRNAs (**right**). Red dots represent significantly upregulated transcripts, and green dots represent downregulated transcripts. (**C**) KEGG pathway enrichment analysis of upregulated (**left**) and downregulated (**right**) differentially expressed genes. The x-axis represents the rich factor; bubble size indicates the number of genes; and color intensity indicates the *p*-value. (**D**) Co-expression network of differentially expressed lncRNAs and their targeted mRNAs in granulosa cells.

**Figure 9:**
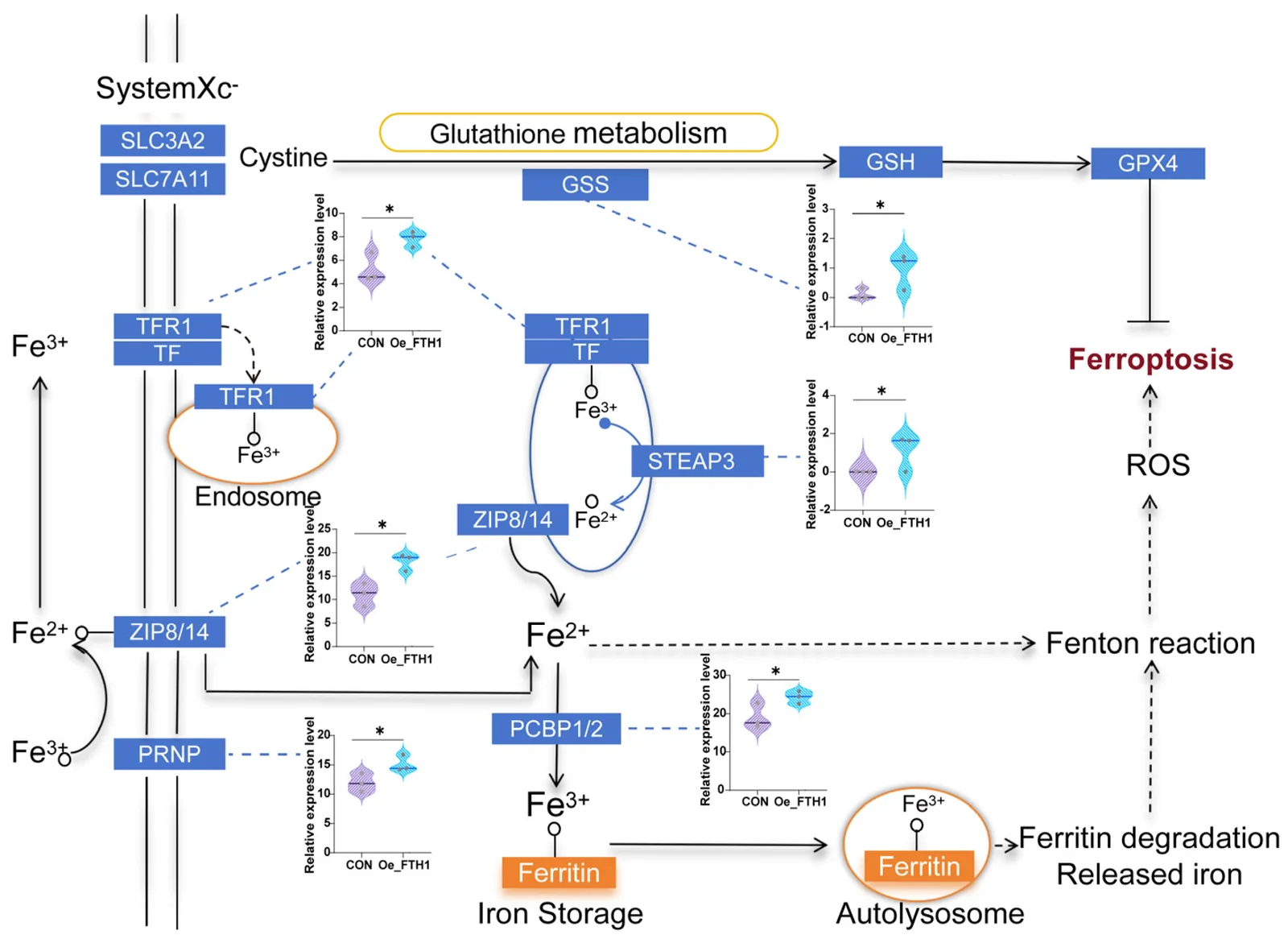
Ferroptosis-related transcriptional changes. Erastin inhibits system xc^−^-mediated cystine uptake, thereby impairing the GSH–GPX4 antioxidant pathway. *FTH1* acts at the ferritin-dependent iron-storage node by sequestering iron and limiting the labile Fe^2+^ pool available for the Fenton reaction. Fer-1 suppresses the downstream propagation of lipid peroxidation and ferroptosis. Violin plots show the relative mRNA expression of *GSS*, *TFR1*, *STEAP3*, *ZIP8/14* and *PCBP1/2* in *FTH1*-overexpressing and control GCs under 10 μM erastin treatment. Data are presented as biological replicate-level values; * *p* < 0.05. Dashed connector lines without arrowheads (shown in blue in the colour version) link the iron-handling genes to their corresponding expression plots; pathway relationships are indicated by directional arrows.

Principal component analysis showed a clear separation between the *FTH1* overexpression and control groups (Figure 8A). Differential expression analysis identified 137 upregulated and 160 downregulated mRNAs, with *FTH1* being the most significantly upregulated gene, confirming the effectiveness of the overexpression model. In parallel, 51 lncRNAs were upregulated, and 41 lncRNAs were downregulated (Figure 8B). KEGG enrichment analysis showed that upregulated genes were enriched in pathways including cytokine-cytokine receptor interaction, protein processing in the endoplasmic reticulum, and the JAK-STAT signaling pathway. Apoptosis, necroptosis and mitophagy–animal were also identified among the pathways enriched by the upregulated DEGs (Figure 8C). Downregulated genes were enriched in oxidative phosphorylation and ATP-dependent chromatin remodeling (Figure 8C). Because *FTH1* attenuated ferroptosis in GCs, we further analyzed ferroptosis-related transcripts (Supplementary Figure S8). Several genes involved in glutathione metabolism and iron handling, including *GSS*, *TFR1*, *PCBP1/2*, *STEAP3* and *ZIP8/14*, were upregulated in *FTH1*-overexpressing cells (Figure 9).

Candidate lncRNA–mRNA associations were explored using Pearson correlation analysis across the six RNA-seq samples. Fourteen differentially expressed lncRNAs and seven differentially expressed mRNAs met the predefined correlation criteria (|r| ≥ 0.60, nominal *p* < 0.05; Figure 8D). ENSOARG00020039947 was positively correlated with several lncRNAs, including MSTRG.9749.10, MSTRG.9749.26 and MSTRG.9749.1, whereas ENSOART00020078118 was negatively correlated with PLEKHN1. XR_003589363.2 and RPS28 showed the strongest correlation. Computational prediction also identified potential miR-29b-1 and miR-29 binding sites in ENSOARG00020033611. In addition, 23 lncRNAs showed expression correlations with *FTH1*, including 16 positive and seven negative correlations (Table 3). ENSOART00020065009 (r = 0.980, *p* = 0.001) and MSTRG.9749.24 (r = 0.971, *p* = 0.001) showed the strongest positive correlations. These results identify candidate co-expression associations for further investigation but do not demonstrate direct regulatory relationships.

## 4. Discussion

Ferroptosis has emerged as an important mechanism linking iron metabolism, oxidative stress and reproductive dysfunction. Recent studies have shown that ferroptosis contributes to follicular atresia and granulosa cell injury in livestock and poultry, including porcine and avian models [16,17,18]. Our previous single-cell analysis further suggested that ferroptosis-related pathways differ between Hu sheep with different litter sizes and identified *FTH1* as a candidate gene associated with GC function [29]. The present study extends these observations by functionally validating *FTH1* in primary sheep GCs. The major finding is that *FTH1* protects GCs from erastin-induced ferroptosis-associated injury, preserves steroidogenic marker expression, and is associated with transcriptional changes in cytokine signaling, ROS-related pathways and lncRNA regulatory networks.

### 4.1. FTH1 Protects Sheep GCs from Erastin-Induced Ferroptosis

*FTH1* expression was higher in GCs from healthy follicles than in those from atretic follicles and was also higher in large follicles than in small follicles. These expression patterns are consistent with recent studies in chickens and sheep showing reduced *FTH1* expression or altered ferroptosis signatures in atretic follicles [17,18]. Collectively, these observations link *FTH1* expression to follicular status and identify it as a candidate marker of GC condition. Because expression differences alone cannot determine whether altered *FTH1* is a cause or consequence of follicular condition, we further examined its functional contribution by manipulating *FTH1* expression in cultured sheep GCs.

The erastin screening experiment provided an important basis for interpreting the functional assays. At 10 μM, erastin induced biochemical stress without markedly reducing cell viability, whereas 20 μM produced stronger ferroptosis-associated injury accompanied by impaired viability and increased oxidative damage. Therefore, 10 μM was used to examine *FTH1*-mediated regulation during mild erastin-induced stress, whereas 20 μM was used to assess its effects during more severe ferroptosis-associated injury [19]. This distinction is important because the effects of *FTH1* were detected at different intensities under the two conditions. At 10 μM, *FTH1* overexpression mainly altered the expression of *GPX4, ACSL4* and *NCOA4*, whereas its effects on MDA and GSH were limited. Under the stronger 20 μM challenge, *FTH1* still reduced Fe^2+^ accumulation and oxidative damage, but its effects on several ferroptosis-related genes were weaker. MDA and GSH reflect accumulated lipid peroxidation and antioxidant consumption, whereas mRNA abundance represents regulation at the selected sampling time [23,48]. Thus, the transcriptional and biochemical responses observed at the two erastin concentrations reflect different stages and regulatory levels of ferroptosis stress rather than inconsistent effects of *FTH1*. Mechanistically, this protective effect is consistent with the core function of *FTH1* in iron storage. *FTH1* oxidizes soluble Fe^2+^ to Fe^3+^ and facilitates iron sequestration within ferritin, thereby limiting the labile iron pool and reducing Fenton reaction-driven ROS production [49,50]. In our study, without erastin stimulation, *FTH1* overexpression significantly elevated intracellular GSH levels while decreasing Fe^2+^ and MDA accumulation, whereas *FTH1* depletion resulted in the opposite alterations. Upon erastin-induced ferroptosis, *FTH1* expression modulation consistently affected ROS production, as well as Fe^2+^, GSH, and MDA levels, with *FTH1* overexpression alleviating and *FTH1* deficiency exacerbating these ferroptosis-associated changes. It is also worth noting that under 10 μM erastin treatment, *FTH1* knockdown increased Fe^2+^ levels but decreased total iron, potentially reflecting distinct changes in iron speciation and overall cellular iron burden. The increase in Fe^2+^ may result from impaired *FTH1*-mediated iron oxidation and storage, whereas the decrease in total iron may involve compensatory changes in iron uptake or export [51]. However, iron uptake and export were not directly assessed in the present study, and the underlying mechanisms require further investigation. These findings suggest that *FTH1* attenuates ferroptosis-associated injury in GCs following erastin exposure, with its protective effects associated with reduced intracellular Fe^2+^ accumulation and improved antioxidant defenses. Although the combined findings support ferroptosis-associated injury, iron dependence was not directly tested using an iron-chelator rescue experiment. Similar protective roles of *FTH1* have been reported in other ferroptosis-related models, although the direction and magnitude of *FTH1* action may vary with cell type, injury intensity and disease context [52].

Previous studies have also identified *FTH1* as a protective regulator of ferroptosis. In PC-12 cells, *FTH1* knockdown reduced cell viability and induced mitochondrial dysfunction, whereas *FTH1* overexpression inhibited NCOA4-mediated ferritinophagy and attenuated ferroptosis-induced cell death [53]. Similarly, *FTH1* overexpression weakened baicalin-induced ferroptosis in bladder cancer cells, thereby reducing the anticancer efficacy of baicalin [54]. *FTH1* is also essential for cellular homeostasis, as its deletion causes embryonic lethality in mice [55], and its silencing promotes protein misfolding and ROS accumulation [56]. However, the function of *FTH1* may be context-dependent. In granulosa cells exposed to follicular fluid from patients with endometriosis, *FTH1* upregulation may represent a compensatory response to iron overload, but is insufficient to prevent ferroptosis-related impairment of oocyte maturation [57]. Together, these findings support the protective role of *FTH1* against ferroptosis, while suggesting that its efficacy depends on the severity of iron stress and the cellular microenvironment.

Taken together, the follicular expression patterns associate *FTH1* with follicular status, whereas the gain- and loss-of-function experiments demonstrate that *FTH1* modifies the susceptibility of cultured sheep GCs to erastin-induced ferroptosis. These findings establish a protective role for *FTH1* in the present cell model, while its contribution to follicular development and atresia in vivo remains to be determined.

### 4.2. FTH1 Preserves Steroidogenic Function During Ferroptosis Stress

A key contribution of this study is that it links *FTH1*-mediated ferroptosis control to GC steroidogenic function. *STAR*, *CYP11A1*, *HSD17B1* and *3β-HSD* encode essential components of the steroidogenic pathway and are closely associated with follicular growth and oocyte maturation [58]. LH and FSH coordinate steroidogenesis in theca cells and GCs, thereby supporting the endocrine microenvironment required for follicular growth and development [59], and FSH promotes estrogen production in GCs. And genes such as *CYP11A1*, *STAR*, and *3β-HSD* control E_2_ and P_4_ synthesis related to reproduction and development in animals [60,61].

In the present study, *FTH1* overexpression increased *STAR* and *CYP11A1* expression under erastin stress, whereas *FTH1* interference reduced *STAR*, *CYP11A1, HSD17B1* and *3β-HSD*. In the absence of erastin, *FTH1* overexpression did not significantly alter E_2_ or P_4_ concentrations. However, under erastin exposure, both hormone concentrations were higher in the *FTH1*-overexpressing group than in the corresponding erastin-treated controls. These findings suggest that *FTH1* attenuates the impairment of GC steroidogenic function during ferroptosis stress rather than directly stimulating basal steroid hormone production.

The preservation of P_4_ production may be explained partly by protection of the mitochondrial steps of steroidogenesis. *STAR* transports cholesterol to the inner mitochondrial membrane [62], where *CYP11A1* converts cholesterol to pregnenolone [63], which is subsequently converted to P_4_ by *3β-HSD*. Ferroptosis stress is associated with mitochondrial structural injury [64]; therefore, the reductions in Fe^2+^ accumulation, ROS production and lipid peroxidation following *FTH1* overexpression may help maintain STAR–CYP11A1 activity and the early steroidogenic pathway required for P_4_ production.

The E_2_ response involves a related but distinct steroidogenic route. According to the two-cell, two-gonadotrophin model, androgen substrates produced by theca cells are transferred to GCs and converted into estrogens through the granulosa-cell estrogenic pathway [65]. In the present study, *FTH1* overexpression was associated with higher E_2_ concentrations under erastin treatment, whereas *FTH1* interference reduced *HSD17B1* expression and E_2_ production. However, *CYP19A1* expression and the availability of androgen substrates were not assessed, and the isolated GC model did not reproduce the physiological supply of substrates from theca cells. Therefore, these findings indicate that *FTH1* attenuated the erastin-associated reduction in E_2_ production under the present culture conditions, rather than demonstrating direct regulation of the complete estrogen biosynthetic pathway.

Iron-dependent oxidative injury provides a plausible upstream connection between ferroptosis and both steroidogenic routes. Iron overload has been shown to increase ROS accumulation and reduce GC viability while suppressing *CYP19A1*, the aromatase required for estrogen biosynthesis [66]. By limiting the labile iron pool and oxidative damage, *FTH1* may therefore maintain both the mitochondrial machinery supporting P_4_ synthesis and the granulosa-cell enzymatic capacity required for E_2_ production. Consistently, Fer-1 treatment was associated with higher E_2_ and P_4_ concentrations than erastin treatment alone, further linking ferroptosis inhibition with preservation of GC endocrine function.

However, the CCK-8 assay used in the present study reflects cellular metabolic activity and viability rather than cell proliferation. Therefore, whether *FTH1* directly regulates GC proliferation remains unclear and should be examined using EdU incorporation, Ki-67 expression or cell-cycle analysis. This distinction is relevant because proliferation, apoptosis and E_2_ secretion are coordinately regulated during sheep follicular development [67].

Collectively, these results support a protective association between *FTH1*-mediated resistance to ferroptosis-associated injury and the maintenance of GC endocrine function. The hormone changes may arise indirectly from the preservation of cellular viability, redox homeostasis and steroidogenic machinery under erastin stress; they do not demonstrate direct transcriptional or enzymatic regulation of steroidogenesis by *FTH1*.

### 4.3. Erastin-Triggered Ferroptosis Is Associated with Apoptotic Injury in GCs

GC apoptosis is a central event during follicular atresia [12]. However, increasing evidence suggests that apoptosis and ferroptosis are not isolated processes in ovarian follicles. Ferroptosis-related genes and metabolic changes have been implicated in GC dysfunction [68,69]. A study has found that abnormal glutathione metabolism exists in the porcine follicular atresia process, suggesting that ferroptosis plays an important regulatory role in follicular atresia [70]. Another study investigating goose follicle development and selection found that follicular fluid exosomes mediate the ferroptosis pathway to induce programmed cell death of GCs and participate in the regulation of follicular atresia [26]. In our study, Fer-1 treatment was associated with improved cell viability and steroidogenic marker expression, together with a lower apoptotic cell percentage and reduced CASP3 expression during erastin exposure. Although ferroptosis and apoptosis are executed through distinct molecular pathways, they share several upstream stress signals. Iron accumulation, ROS production, GSH depletion, lipid peroxidation and mitochondrial dysfunction can promote ferroptotic injury and increase cellular susceptibility to caspase-dependent apoptosis [71]. The coordinated changes observed after Fer-1 treatment therefore support an association between ferroptosis stress and apoptotic injury in sheep GCs. However, the present experiments do not establish whether ferroptosis directly activates apoptosis or determine the temporal sequence of these processes. Further assessment of mitochondrial function, lipid-peroxidation products and caspase activity will be required to define their mechanistic relationship. Within these limits, the *FTH1*–ferroptosis axis may influence follicular fate by reducing ferroptosis-associated oxidative injury and the associated apoptotic response.

### 4.4. Exploratory mRNA–lncRNA Associations Related to FTH1-Mediated Protection

RNA-seq analysis was performed to explore transcriptional changes associated with *FTH1* overexpression under erastin-induced stress. Because the sequencing design compared 10 μM erastin-treated GCs with and without *FTH1* overexpression, the results should be interpreted as *FTH1*-associated changes under ferroptosis stress rather than as the complete baseline regulatory network of *FTH1*. Within this context, *FTH1* overexpression caused a clear shift in the transcriptomic landscape. Upregulated genes were enriched in cytokine-cytokine receptor interaction, protein processing in the endoplasmic reticulum and JAK-STAT signaling, whereas downregulated genes were enriched in oxidative phosphorylation and ROS-related pathways. Among the upregulated genes, *SOCS2*, *NGF* and *TGF-β3* are involved in hormone responsiveness, GC function and follicular development. *SOCS2* acts as a feedback inhibitor of the JAK/STAT signaling pathway and is involved in modulating hormone responsiveness during female reproduction [72]. NGF is also essential for ovarian follicular development, regulating primordial follicle assembly, ovulation, and granulosa cell endocrine function through its high-affinity receptor TrkA, partly by promoting *FSHR* and *LHCGR* expression as well as progesterone and estrogen secretion [73]. In addition, TGF-β3, a member of the TGF-β superfamily, promotes granulosa cell proliferation and differentiation [74], and its higher abundance in large follicular fluid further supports its positive role in follicle development and oocyte maturation [75]. In contrast, CYBA, a component required for NADPH oxidase activity and NOX-mediated ROS generation [19,76], was downregulated. These changes are consistent with the observed reduction in ROS accumulation after *FTH1* overexpression and support the idea that *FTH1* reshapes both iron-redox balance and paracrine signaling under ferroptosis stress.

*TFR1* mediates transferrin-bound iron uptake and can influence ferroptosis sensitivity by regulating cellular iron availability [77]. Its increased expression following *FTH1* overexpression may appear inconsistent with the observed reduction in intracellular Fe^2+^. One possible explanation is a compensatory response to *FTH1*-mediated iron sequestration: a reduction in the labile iron pool can stabilize *TFR1* mRNA through the IRP–IRE system, thereby maintaining cellular iron acquisition [78]. Thus, the increase in *TFR1* expression may reflect a rebalancing between iron uptake and ferritin storage rather than increased accumulation of redox-active iron. Because TFR1 protein abundance, iron-uptake activity and IRP binding were not assessed, this interpretation requires further validation.

The additional enrichment of apoptosis, necroptosis and mitophagy-related genes broadens the transcriptomic context of *FTH1* action under erastin-induced stress. Although apoptosis, necroptosis and ferroptosis are executed through distinct molecular machinery, interactions among these regulated cell-death programs can influence cellular responses to stress [79]. The apoptosis-related enrichment is consistent with our observation that erastin-induced ferroptotic damage was accompanied by increased apoptosis, whereas Fer-1 reduced both oxidative injury and apoptotic indices. This association supports the presence of shared stress responses but does not indicate a direct conversion of ferroptosis into apoptosis. The enrichment of mitophagy may further reflect mitochondrial quality control in response to oxidative and iron-related stress. BNIP3/NIX-mediated mitophagy has been shown to restrict ferroptosis by removing mitochondria that produce excessive ROS [80]. Conversely, enhanced mitophagy can also increase the availability of labile iron and promote ferroptosis under specific conditions [81]. Therefore, the enrichment of apoptosis, necroptosis and mitophagy should be interpreted as *FTH1*-associated transcriptional remodelling of interconnected stress-response pathways rather than evidence that these pathways were functionally activated in sheep GCs. Further assessment of caspase activity, RIPK3–MLKL signaling and mitophagy flux will be required to define their respective contributions.

The lncRNA analysis further suggested a potential regulatory layer upstream or downstream of *FTH1*. LncRNAs are increasingly recognized as regulators of female reproduction and ferroptosis-related cellular injury [82,83,84]. In this study, ENSOART00020065009 and MSTRG.9749.24 showed strong positive correlations with *FTH1*, whereas several other lncRNAs were negatively correlated with *FTH1* expression. In addition, predicted interactions involving miR-29b-1 and miR-29 indicate that lncRNA-miRNA-mRNA regulatory crosstalk may participate in *FTH1*-associated ferroptosis regulation. This prediction is biologically plausible because miR-29a-5p has been shown to regulate ferroptosis by directly targeting *FTH1* in another cellular model [85]. *FTH1* expression can also be regulated at the epitranscriptomic level. *FTO*-dependent m^6^A modification and the m6A readers *YTHDF1* and *YTHDF2* have been reported to control *FTH1* mRNA abundance and ferroptosis sensitivity [86]. In addition, ferritin heavy chain has been linked to *DNMT1*-dependent methylation of the miR-125b promoter and subsequent regulation of *p53*-mediated apoptosis [87]. These findings identify miRNA activity, RNA methylation and DNA methylation as potential intracellular mediators of *FTH1* action.

Overall, the exploratory transcriptomic analysis associated *FTH1* overexpression with changes in cytokine signaling, redox-related pathways and non-coding RNA expression under erastin-induced stress. The predicted lncRNA–mRNA relationships are based on expression correlations and computational target prediction and should therefore be considered candidate associations rather than established regulatory pathways. Together, these reports raise the possibility that miRNA-mediated regulation and RNA or DNA methylation may influence *FTH1* expression in other cellular contexts. Because these processes were not examined in the present study, their relevance to the *FTH1* response in sheep GCs remains to be established.

### 4.5. Potential Biological Implications and Future Validation

The association of *FTH1* with GC survival and steroid hormone production suggests that *FTH1* may be involved in maintaining granulosa cell function under ferroptotic stress. However, the present findings are based mainly on granulosa cell models and should not be interpreted as evidence for direct reproductive or veterinary application. Although *FTH1* may be considered together with ferroptosis-related indicators such as *GPX4*, *SLC7A11*, Fe^2+^, MDA, and GSH in future studies of follicular injury, its diagnostic or predictive value remains unvalidated. Similarly, the protective effects of Fer-1 observed in vitro indicate that ferroptosis-related pathways deserve further investigation. However, all endpoints were assessed after a single treatment duration. Biochemical and endocrine changes were detectable at 10 μM erastin, whereas overt morphological injury and apoptosis were mainly observed at 20 μM. Because steroidogenic enzyme activity, CASP3 cleavage and caspase activity were not measured, we cannot determine whether this difference reflects temporal regulation, post-transcriptional control or a nonlinear threshold for cellular injury. Therefore, they do not yet support the use of ferroptosis-targeting strategies in reproductive management. Further studies using naturally atretic follicles, ovarian tissue models, in vivo animal experiments, and independent sheep populations are required to determine whether these findings have physiological relevance at the follicle, ovary, and animal level.

## 5. Conclusions

*FTH1* expression was associated with follicular status, and functional experiments further showed that *FTH1* influenced the response of cultured sheep GCs to erastin-induced ferroptosis-associated injury. *FTH1* overexpression limited Fe^2+^ accumulation, ROS production and lipid peroxidation, supported the GSH–GPX4 antioxidant system, and attenuated the reductions in steroidogenic markers and E_2_ and P_4_ concentrations under the present culture conditions. *FTH1* interference generally produced the opposite effects. The attenuation of erastin-associated injury by Fer-1 was accompanied by reduced apoptosis and CASP3 expression, supporting potential crosstalk between ferroptosis and apoptosis without establishing a causal sequence. The RNA-seq analysis was also exploratory, with three biological replicates per group and no corresponding untreated transcriptomic controls, limiting the statistical power and preventing separation of basal *FTH1* effects from stress-dependent responses. At the same time, exploratory transcriptomic analysis further linked *FTH1* overexpression to changes in cytokine- and ROS-related pathways and identified candidate lncRNA–mRNA co-expression relationships requiring functional validation. Collectively, these findings identify *FTH1* as a candidate marker of follicular condition and support its protective role against ferroptosis-associated injury in cultured sheep GCs, providing a basis for further validation in follicular and in vivo models.

## Figures and Tables

**Table 1:** Information on *FTH1* siRNA.

Name	Sequences (5′-3′)
siRNA-1	Sense: GCUACUGGAACUGCACAAATT
	Antisense: UUUGUGCAGUUCCAGUAGCTT
siRNA-2	Sense: GCAUGGCAGAGUACCUCUUTT
	Antisense: AAGAGGUACUCUGCCAUGCTT
Negative control (NC)	Sense: UUCUCCGAACGUGUCACGUTT
	Antisense: ACGUGACACGUUCGGAGAATT

**Table 2:** RT-qPCR primer information.

Gene Name	Sequences (5’-3’)	Product Size (bp)	References
*Beta actin*	F: ACCGTGAGAAGATGACCCAGA	92	[37]
	R: AGAGGCGTACAGGGACAGCA		
*FTH1*	F: ATGCAATGGAATGTGCGCTG	145	-
	R: TTGATGGCTTCCACCTGCTC		
*ACSL4*	F: CAGCGCCTCGGATTCAAA	172	-
	R: GAAGGCAATAATGGGAGCAGC		
*GPX4*	F: GTTCGCTGCTGGCTATAACG	137	[38]
	R: TCCATTTGATGGCGTTTCCC		
*NCOA4*	F: TTCAGAGCAGTCATTGCGGT	130	[38]
	R: ACAATGGCAAACGTGGCATC		
*SLC7A11*	F: GTTCTGTCACTGTTTGGAGCC	186	[38]
	R: ATAGCGTCCAAATGCCAGAG		
*CASP3*	F: TGTGGTATTGAGACAGACAGTG	152	[39]
	R: CAAAGAGCCTGGATGAACC		
*STAR*	F: CATTGACCTCAAGGGATGGCT	131	-
	R: GCCTTCAACACCTGGCTTCA		
*CYP11A1*	F: GTTTCGCTTTGCCTTTGAGTC	158	[40]
	R: ACAGTTCTGGAGGGAGGTTGA		
*HSD17B1*	F: AGTGGTACTCATCACCGGCT	103	-
	R: CCTCAGCGTGGCATACACTTT		
*3 beta HSD*	F: GGAGACATTCTGGATGAGCAG	200	[41]
	R: TCTATGGTGCTGGTGTGGA		

F denotes the forward primer, and R denotes the reverse primer.

**Table 3:** *FTH1*-targeting lncRNAs via trans-regulation.

LncRNA ID	LncRNA Regulate	Type	Target Gene Regulate	Corr	*p* Value
ENSOART00020050195	down	known	up	−0.907	0.013
ENSOART00020042374	up	known	up	0.952	0.003
ENSOART00020065009	up	known	up	0.980	0.001
ENSOART00020055519	up	known	up	0.844	0.035
ENSOART00020079749	down	known	up	−0.873	0.023
ENSOART00020064139	up	known	up	0.875	0.022
XR_003589363.2	down	known	up	−0.845	0.034
MSTRG.9749.3	up	novel	up	0.903	0.014
MSTRG.14107.3	down	novel	up	−0.926	0.008
MSTRG.9749.9	up	novel	up	0.893	0.017
MSTRG.13586.1	down	novel	up	−0.848	0.033
MSTRG.3740.5	up	novel	up	0.815	0.048
MSTRG.11962.1	up	novel	up	0.851	0.032
MSTRG.1506.1	down	novel	up	−0.832	0.040
MSTRG.9749.26	up	novel	up	0.842	0.035
MSTRG.7335.2	up	novel	up	0.900	0.015
MSTRG.9749.24	up	novel	up	0.971	0.001
MSTRG.13454.1	up	novel	up	0.840	0.036
MSTRG.9988.1	up	novel	up	0.926	0.008
MSTRG.3263.1	up	novel	up	0.846	0.034
MSTRG.9746.4	up	novel	up	0.901	0.014
MSTRG.11625.1	down	novel	up	−0.818	0.046
MSTRG.2392.4	up	novel	up	0.830	0.041

## Data Availability

RNA-seq data have been submitted to the China National Center for Bioinformation (CNCB) at the following URL: https://ngdc.cncb.ac.cn/gsa/s/qLC8c2yl (accessed on 1 September 2026).

## Supplementary Materials

The following supporting information can be downloaded at https://www.mdpi.com/article/10.3390/biom16101507/s1: Table S1: The CDS region sequence of *FTH1*; Table S2: RT-qPCR reaction system; Table S3: RT-qPCR reaction program; Table S4: Detection of amplification efficiency of primers; Table S5: Summary of RNA-seq data quality; Table S6: Summary of read mapping statistics; Table S7: The number of detected genes; Figure S1: Morphological and biological identification of healthy follicles and atretic follicles; Figure S2: Isolation and identification of primary granulosa cells from Hu sheep ovaries; Figure S3: Erastin reduces estradiol and progesterone concentrations in sheep granulosa-cell cultures; Figure S4: *FTH1* transfection conditions selection; Figure S5: Selection of *FTH1*-targeting siRNA in sheep granulosa cells. Figure S6: protein-level validation of *FTH1* overexpression and interference; Figure S7: Effects of *FTH1* on ferroptosis-related biochemical indicators without erastin. Figure S8: Expression changes in ferroptosis-pathway genes after *FTH1* overexpression under erastin stress.

## Abbreviations

The following abbreviations are used in this manuscript:

TFR1	transferrin receptor 1
Fer-1	Ferrostatin-1
FTH1	ferritin heavy chain 1
NCOA4	nuclear receptor coactivator 4
GC	granulosa cell
ROS	reactive oxygen species
DMSO	dimethyl sulfoxide
RT-qPCR	reverse transcription quantitative polymerase chain reaction
GPX4	glutathione peroxidase 4
GAPDH	glyceraldehyde-3-phosphate dehydrogenase
CYP11A1	Cytochrome P450, family 11, subfamily A, polypeptide 1
STAR	steroidogenic acute regulatory protein
CCK-8	Cell Counting Kit-8
KEGG	Kyoto Encyclopedia of Genes and Genomes
LF	large follicles
SF	small follicles
HF	healthy follicles
AF	atretic follicles
HSD17B1	hydroxysteroid 17-beta dehydrogenase 1
3β-HSD	3β-hydroxysteroid dehydrogenase
ACSL4	acyl-CoA Synthetase long-chain family member 4
MDA	malondialdehyde
GSH	reduced glutathione
lncRNA	long non-coding RNA
SLC7A11	solute carrier family 7 member 11
CASP3	caspase 3
ZIP8/ZIP14	Zrt- and Irt-like protein 8 / 14
PCBP1/PCBP2	poly(rC)-binding protein 1 / 2
SOCS2	suppressor of cytokine signaling 2
NGF	nerve growth factor
TGF-β	transforming growth factor beta
E2	estradiol (17β-estradiol)
P4	progesterone
FBS	fetal bovine serum
DMEM/F12	Dulbecco’s modified Eagle medium/Ham’s nutrient mixture F-12

## References

[1] Zhang R., Liu Y., Li W., Wang P., Liu Z., Wen Y., Chu M., Wang L. (2024). A Mutation in *LPAR2* Activates the miR-939-5p-LPAR2-PI3K/AKT Axis to Regulate the Proliferation and Apoptosis of Granulosa Cells in Sheep. Theriogenology.

[2] Han S., Wei Y., Wei Y., Zhao X., Chen Y., Cui C., Zhang Y., Yin H. (2025). Mechanisms of Programmed Cell Death in Livestock Follicular Development and Atresia: A Review. J. Anim. Sci. Biotechnol..

[3] Jiang A., Li J., Wang L., Liu Y., Wu Z., Wang H., Wu S., Bao W. (2025). HIF1A Regulates Follicular Atresia through O-GlcNAcylation-Mediated VEZF1/ET-1/FOXO1/BAX Signaling in Porcine Granulosa Cells. J. Anim. Sci. Biotechnol..

[4] Bao Y., Li X., El-Samahy M.A., Yang H., Wang Z., Yang F., Yao X., Wang F. (2023). Exploration the Role of INHBA in Hu Sheep Granulosa Cells Using RNA-Seq. Theriogenology.

[5] Lu C., Yang W., Hu Z., Liu Y. (2005). Granulosa Cell Proliferation Differentiation and Its Role in Follicular Development. Chin. Sci. Bull..

[6] Baena V., Terasaki M. (2019). Three-Dimensional Organization of Transzonal Projections and Other Cytoplasmic Extensions in the Mouse Ovarian Follicle. Sci. Rep..

[7] Stocco D.M. (2001). StAR Protein and the Regulation of Steroid Hormone Biosynthesis. Annu. Rev. Physiol..

[8] Miller W.L. (2013). Steroid Hormone Synthesis in Mitochondria. Mol. Cell. Endocrinol..

[9] Rasmussen M.K., Ekstrand B., Zamaratskaia G. (2013). Regulation of 3β-Hydroxysteroid Dehydrogenase/Δ^5^-Δ^4^ Isomerase: A Review. Int. J. Mol. Sci..

[10] Mindnich R., Möller G., Adamski J. (2004). The Role of 17 Beta-Hydroxysteroid Dehydrogenases. Mol. Cell. Endocrinol..

[11] Matsuda F., Inoue N., Manabe N., Ohkura S. (2012). Follicular Growth and Atresia in Mammalian Ovaries: Regulation by Survival and Death of Granulosa Cells. J. Reprod. Dev..

[12] Inoue N., Matsuda F., Goto Y., Manabe N. (2011). Role of Cell-Death Ligand-Receptor System of Granulosa Cells in Selective Follicular Atresia in Porcine Ovary. J. Reprod. Dev..

[13] Boone D.L., Tsang B.K. (1998). Caspase-3 in the Rat Ovary: Localization and Possible Role in Follicular Atresia and Luteal Regression. Biol. Reprod..

[14] Chen H., Nie P., Li J., Wu Y., Yao B., Yang Y., Lash G.E., Li P. (2024). Cyclophosphamide Induces Ovarian Granulosa Cell Ferroptosis via a Mechanism Associated with HO-1 and ROS-Mediated Mitochondrial Dysfunction. J. Ovarian Res..

[15] Voros C., Chatzinikolaou F., Papadimas G., Polykalas S., Mavrogianni D., Koulakmanidis A.-M., Athanasiou D., Kanaka V., Bananis K., Athanasiou A. (2026). Oxidative Stress and SIRT1-Nrf2 Anti-Ferroptotic Pathways in Granulosa Cells: A Molecular Key to Follicular Atresia and Ovarian Aging. Int. J. Mol. Sci..

[16] Yang Y., He Y., Gan M., Zhao X., Liao T., Lei Y., Chen L., Niu L., Zhao Y., Wang Y. (2025). The Mechanism of Ferroptosis Regulating Granulosa Cell Apoptosis and Oxidative Stress Through the NF-κB/PTGS2 Axis in Porcine Atretic Follicles. Antioxidants.

[17] Song Y., Hai E., He L., Zhang N., Zhang N., Wang J., Sun Y., Zeng D., Zhang J. (2025). Linoleic Acid Metabolite 13-Hydroxyoctadecadienoic Acid as a Biphasic Ferroptosis Modulator in Granulosa Cells: Multi-Omics Analysis of Ovine Atretic Follicles. Front. Cell Dev. Biol..

[18] Han S., Yu C., Qiu M., Xiong X., Peng H., Song X., Hu C., Zhang Z., Xia B., Yang L. (2024). USP13 Regulates Ferroptosis in Chicken Follicle Granulosa Cells by Deubiquitinating ATG7. Poult. Sci..

[19] Dixon S.J., Lemberg K.M., Lamprecht M.R., Skouta R., Zaitsev E.M., Gleason C.E., Patel D.N., Bauer A.J., Cantley A.M., Yang W.S. (2012). Ferroptosis: An Iron-Dependent Form of Nonapoptotic Cell Death. Cell.

[20] Doll S., Proneth B., Tyurina Y.Y., Panzilius E., Kobayashi S., Ingold I., Irmler M., Beckers J., Aichler M., Walch A. (2017). ACSL4 Dictates Ferroptosis Sensitivity by Shaping Cellular Lipid Composition. Nat. Chem. Biol..

[21] Stockwell B.R., Friedmann Angeli J.P., Bayir H., Bush A.I., Conrad M., Dixon S.J., Fulda S., Gascón S., Hatzios S.K., Kagan V.E. (2017). Ferroptosis: A Regulated Cell Death Nexus Linking Metabolism, Redox Biology, and Disease. Cell.

[22] Ayala A., Muñoz M.F., Argüelles S. (2014). Lipid Peroxidation: Production, Metabolism, and Signaling Mechanisms of Malondialdehyde and 4-Hydroxy-2-Nonenal. Oxid. Med. Cell. Longev..

[23] Yang W.S., SriRamaratnam R., Welsch M.E., Shimada K., Skouta R., Viswanathan V.S., Cheah J.H., Clemons P.A., Shamji A.F., Clish C.B. (2014). Regulation of Ferroptotic Cancer Cell Death by GPX4. Cell.

[24] Jiang X., Stockwell B.R., Conrad M. (2021). Ferroptosis: Mechanisms, Biology, and Role in Disease. Nat. Rev. Mol. Cell Biol..

[25] Dixon S.J., Olzmann J.A. (2024). The Cell Biology of Ferroptosis. Nat. Rev. Mol. Cell Biol..

[26] Zhang Y., Jiang Y., Dong X., Luo S., Jiao G., Weng K., Bao Q., Zhang Y., Vongsangnak W., Chen G. (2024). Follicular Fluid-Derived Exosomal HMOX1 Promotes Granulosa Cell Ferroptosis Involved in Follicular Atresia in Geese (*Anser cygnoides*). Poult. Sci..

[27] Deng Y., Zeng L., Liu H., Zuo A., Zhou J., Yang Y., You Y., Zhou X., Peng B., Lu H. (2024). Silibinin Attenuates Ferroptosis in Acute Kidney Injury by Targeting FTH1. Redox Biol..

[28] Latunde-Dada G.O. (2017). Ferroptosis: Role of Lipid Peroxidation, Iron and Ferritinophagy. Biochim. Biophys. Acta BBA Gen. Subj..

[29] Ge T., Wen Y., Li B., Huang X., Jiang S., Zhang E. (2023). Single-Cell Sequencing Reveals the Reproductive Variations between Primiparous and Multiparous Hu Ewes. J. Anim. Sci. Biotechnol..

[30] Mo J., Sun L., Cheng J., Lu Y., Wei Y., Qin G., Liang J., Lan G. (2021). Non-Targeted Metabolomics Reveals Metabolic Characteristics of Porcine Atretic Follicles. Front. Vet. Sci..

[31] Zhai B., Li X., Zhao Z., Cao Y., Liu X., Liu Z., Ma H., Lu W. (2023). Melatonin Protects the Apoptosis of Sheep Granulosa Cells by Suppressing Oxidative Stress via MAP3K8 and FOS Pathway. Genes.

[32] Jia Y., Liu Y., Wang P., Liu Z., Zhang R., Chu M., Zhao A. (2024). NTRK2 Promotes Sheep Granulosa Cells Proliferation and Reproductive Hormone Secretion and Activates the PI3K/AKT Pathway. Animals.

[33] Wei X., Huang Q., Huang J., Yu L., Chen J. (2023). Erastin Induces Ferroptosis in Cervical Cancer Cells via Nrf2/HO-1 Signaling Pathway. Int. J. Immunopathol. Pharmacol..

[34] Skouta R., Dixon S.J., Wang J., Dunn D.E., Orman M., Shimada K., Rosenberg P.A., Lo D.C., Weinberg J.M., Linkermann A. (2014). Ferrostatins Inhibit Oxidative Lipid Damage and Cell Death in Diverse Disease Models. J. Am. Chem. Soc..

[35] Hou W., Xie Y., Song X., Sun X., Lotze M.T., Zeh H.J., Kang R., Tang D. (2016). Autophagy Promotes Ferroptosis by Degradation of Ferritin. Autophagy.

[36] Koppula P., Zhuang L., Gan B. (2021). Cystine Transporter SLC7A11/xCT in Cancer: Ferroptosis, Nutrient Dependency, and Cancer Therapy. Protein Cell.

[37] Li B., Huang X., Yang C., Ge T., Zhao L., Zhang X., Tian L., Zhang E. (2021). miR-27a Regulates Sheep Adipocyte Differentiation by Targeting CPT1B Gene. Animals.

[38] Wang L., Yang F., Hu M., Chen G., Wang Y., Xue H., Fu D., Bai H., Hu G., Cao H. (2023). GPX4 Utilization by Selenium Is Required to Alleviate Cadmium-Induced Ferroptosis and Pyroptosis in Sheep Kidney. Environ. Toxicol..

[39] Zhang L., Sun Y., Jiang C., Sohail T., Sun X., Wang J., Li Y. (2024). Damage to Mitochondria During the Cryopreservation, Causing ROS Leakage, Leading to Oxidative Stress and Decreased Quality of Ram Sperm. Reprod. Domest. Anim..

[40] Wang Y., Duan C., Guo Y., Li J., He H., Li R., Zhang Y., Liu Y. (2022). Effects of Glucose on Glycolysis and Steroidogenesis as Well as Related Gene Expression in Ovine Granulosa Cells in Vitro. Small Rumin. Res..

[41] Chen C., Lu J., Qu Y., Dong J., Hong Y., Ren Y., Jiang S., Baptista R., Wang D., Mur L.A. (2025). MNQ Derivative D19 Alleviates LPS-Induced Inflammation and Oxidative Stress in Sheep Follicular Granulosa Cells through the GPX4-Mediated Ferroptosis. Front. Vet. Sci..

[42] Likhite N., Warawdekar U.M. (2011). A Unique Method for Isolation and Solubilization of Proteins after Extraction of RNA from Tumor Tissue Using Trizol. J. Biomol. Tech..

[43] Love M.I., Huber W., Anders S. (2014). Moderated Estimation of Fold Change and Dispersion for RNA-Seq Data with DESeq2. Genome Biol..

[44] Pertea M., Pertea G.M., Antonescu C.M., Chang T.-C., Mendell J.T., Salzberg S.L. (2015). StringTie Enables Improved Reconstruction of a Transcriptome from RNA-Seq Reads. Nat. Biotechnol..

[45] Sun L., Luo H., Bu D., Zhao G., Yu K., Zhang C., Liu Y., Chen R., Zhao Y. (2013). Utilizing Sequence Intrinsic Composition to Classify Protein-Coding and Long Non-Coding Transcripts. Nucleic Acids Res..

[46] Kong L., Zhang Y., Ye Z.-Q., Liu X.-Q., Zhao S.-Q., Wei L., Gao G. (2007). CPC: Assess the Protein-Coding Potential of Transcripts Using Sequence Features and Support Vector Machine. Nucleic Acids Res..

[47] Wang L., Park H.J., Dasari S., Wang S., Kocher J.-P., Li W. (2013). CPAT: Coding-Potential Assessment Tool Using an Alignment-Free Logistic Regression Model. Nucleic Acids Res..

[48] Forman H.J., Zhang H., Rinna A. (2009). Glutathione: Overview of Its Protective Roles, Measurement, and Biosynthesis. Mol. Asp. Med..

[49] Vidal R., Ghetti B., Takao M., Brefel-Courbon C., Uro-Coste E., Glazier B.S., Siani V., Benson M.D., Calvas P., Miravalle L. (2004). Intracellular Ferritin Accumulation in Neural and Extraneural Tissue Characterizes a Neurodegenerative Disease Associated with a Mutation in the Ferritin Light Polypeptide Gene. J. Neuropathol. Exp. Neurol..

[50] Hasan M.R., Tosha T., Theil E.C. (2008). Ferritin Contains Less Iron (59Fe) in Cells When the Protein Pores Are Unfolded by Mutation. J. Biol. Chem..

[51] Haschka D., Tymoszuk P., Petzer V., Hilbe R., Heeke S., Dichtl S., Skvortsov S., Demetz E., Berger S., Seifert M. (2021). Ferritin H Deficiency Deteriorates Cellular Iron Handling and Worsens Salmonella Typhimurium Infection by Triggering Hyperinflammation. JCI Insight.

[52] Wang S., Guo Q., Zhou L., Xia X. (2024). Ferroptosis: A Double-Edged Sword. Cell Death Discov..

[53] Tian Y., Lu J., Hao X., Li H., Zhang G., Liu X., Li X., Zhao C., Kuang W., Chen D. (2020). FTH1 Inhibits Ferroptosis Through Ferritinophagy in the 6-OHDA Model of Parkinson’s Disease. Neurotherapeutics.

[54] Kong N., Chen X., Feng J., Duan T., Liu S., Sun X., Chen P., Pan T., Yan L., Jin T. (2021). Baicalin Induces Ferroptosis in Bladder Cancer Cells by Downregulating FTH1. Acta Pharm. Sin. B.

[55] Ferreira C., Bucchini D., Martin M.E., Levi S., Arosio P., Grandchamp B., Beaumont C. (2000). Early Embryonic Lethality of H Ferritin Gene Deletion in Mice. J. Biol. Chem..

[56] Zolea F., Biamonte F., Candeloro P., Di Sanzo M., Cozzi A., Di Vito A., Quaresima B., Lobello N., Trecroci F., Di Fabrizio E. (2015). H Ferritin Silencing Induces Protein Misfolding in K562 Cells: A Raman Analysis. Free Radic. Biol. Med..

[57] Ni Z., Li Y., Song D., Ding J., Mei S., Sun S., Cheng W., Yu J., Zhou L., Kuang Y. (2022). Iron-Overloaded Follicular Fluid Increases the Risk of Endometriosis-Related Infertility by Triggering Granulosa Cell Ferroptosis and Oocyte Dysmaturity. Cell Death Dis..

[58] Wang S., Liu B., Liu W., Xiao Y., Zhang H., Yang L. (2017). The Effects of Melatonin on Bovine Uniparental Embryos Development in Vitro and the Hormone Secretion of COCs. PeerJ.

[59] Mészárosová M., Sirotkin A.V., Grossmann R., Darlak K., Valenzuela F. (2008). The Effect of Obestatin on Porcine Ovarian Granulosa Cells. Anim. Reprod. Sci..

[60] Manna P.R., Dyson M.T., Stocco D.M. (2009). Role of Basic Leucine Zipper Proteins in Transcriptional Regulation of the Steroidogenic Acute Regulatory Protein Gene. Mol. Cell. Endocrinol..

[61] Stocco C., Telleria C., Gibori G. (2007). The Molecular Control of Corpus Luteum Formation, Function, and Regression. Endocr. Rev..

[62] Koganti P.P., Zhao A.H., Kern M.C., Fassinou A.C.R., Selvaraj V. (2025). STAR/STARD1: A Mitochondrial Intermembrane Space Cholesterol Shuttle Degraded through Mitophagy. Proc. Natl. Acad. Sci. USA.

[63] Bose H.S. (2024). Dry Molten Globule Conformational State of CYP11A1 (SCC) Regulates the First Step of Steroidogenesis in the Mitochondrial Matrix. iScience.

[64] Ding X., Cui L., Mi Y., Hu J., Cai Z., Tang Q., Yang L., Yang Z., Wang Q., Li H. (2025). Ferroptosis in Cancer: Revealing the Multifaceted Functions of Mitochondria. Cell. Mol. Life Sci..

[65] Hillier S.G., Whitelaw P.F., Smyth C.D. (1994). Follicular Oestrogen Synthesis: The “two-Cell, Two-Gonadotrophin” Model Revisited. Mol. Cell. Endocrinol..

[66] Huang Z., You Y., Qiu Q., Dong N., Hu X., Cai M., Wu Y., Cao C., Zhang Q. (2025). Iron-Responsive ZNF185 Overexpression Drives Mitochondrial Fission and Endoplasmic Reticulum Stress via Cytoskeletal Remodeling in Granulosa Cells. Cell Death Discov..

[67] Xu H., Cai Y., Yang H., Zhang C., Liu W., Zhao B., Wang F., Zhang Y. (2025). AMH Regulates Granulosa Cell Function via ESR2 / P38-MAPK Signaling Pathway in Sheep. Commun. Biol..

[68] Chen X., Song Q.L., Li Z.H., Ji R., Wang J.Y., Cao M.L., Mu X.F., Zhang Y., Guo D.Y., Yang J. (2023). Pterostilbene Ameliorates Oxidative Damage and Ferroptosis in Human Ovarian Granulosa Cells by Regulating the Nrf2/HO-1 Pathway. Arch. Biochem. Biophys..

[69] Zhang S., Liu Q., Chang M., Pan Y., Yahaya B.H., Liu Y., Lin J. (2023). Chemotherapy Impairs Ovarian Function through Excessive ROS-Induced Ferroptosis. Cell Death Dis..

[70] Zhang J., Liu Y., Yao W., Li Q., Liu H., Pan Z. (2018). Initiation of Follicular Atresia: Gene Networks during Early Atresia in Pig Ovaries. Reproduction.

[71] Wu P., Zhang X., Duan D., Zhao L. (2023). Organelle-Specific Mechanisms in Crosstalk between Apoptosis and Ferroptosis. Oxidative Med. Cell. Longev..

[72] Turnley A.M. (2005). Role of SOCS2 in Growth Hormone Actions. Trends Endocrinol. Metab. TEM.

[73] Wang Y., Liu W., Du J., Yu Y., Liang N., Liang M., Yao G., Cui S., Huang H., Sun F. (2015). NGF Promotes Mouse Granulosa Cell Proliferation by Inhibiting ESR2 Mediated Down-Regulation of CDKN1A. Mol. Cell. Endocrinol..

[74] Liang N., Xu Y., Yin Y., Yao G., Tian H., Wang G., Lian J., Wang Y., Sun F. (2011). Steroidogenic Factor-1 Is Required for TGF-Β3-Mediated 17β-Estradiol Synthesis in Mouse Ovarian Granulosa Cells. Endocrinology.

[75] Lai T.-H., Chen H.-T., Wu P.-H., Wu W.-B. (2024). The Presence of TGFβ3 in Human Ovarian Intrafollicular Fluid and Its Involvement in Thromboxane Generation in Follicular Granulosa Cells through a Canonical TGFβRI, Smad2/3 Signaling Pathway and COX-2 Induction. Int. J. Mol. Sci..

[76] Zhuang S., Fu J., Wu L., Xu X., Guo C. (2025). Role of USF1 in Activating CYBA Transcription and Influencing NADPH-ROS-Mediated Oxidative Stress and Lipid Accumulation in Non-Alcoholic Fatty Liver Disease. Biochim. Biophys. Acta Mol. Cell Biol. Lipids.

[77] Gao M., Monian P., Quadri N., Ramasamy R., Jiang X. (2015). Glutaminolysis and Transferrin Regulate Ferroptosis. Mol. Cell.

[78] Feng H., Schorpp K., Jin J., Yozwiak C.E., Hoffstrom B.G., Decker A.M., Rajbhandari P., Stokes M.E., Bender H.G., Csuka J.M. (2020). Transferrin Receptor Is a Specific Ferroptosis Marker. Cell Rep..

[79] Ai Y., Meng Y., Yan B., Zhou Q., Wang X. (2024). The Biochemical Pathways of Apoptotic, Necroptotic, Pyroptotic, and Ferroptotic Cell Death. Mol. Cell.

[80] Yamashita S., Sugiura Y., Matsuoka Y., Maeda R., Inoue K., Furukawa K., Fukuda T., Chan D.C., Kanki T. (2024). Mitophagy Mediated by BNIP3 and NIX Protects against Ferroptosis by Downregulating Mitochondrial Reactive Oxygen Species. Cell Death Differ..

[81] Yu F., Zhang Q., Liu H., Liu J., Yang S., Luo X., Liu W., Zheng H., Liu Q., Cui Y. (2022). Dynamic O-GlcNAcylation Coordinates Ferritinophagy and Mitophagy to Activate Ferroptosis. Cell Discov..

[82] Chen L.-L., Kim V.N. (2024). Small and Long Non-Coding RNAs: Past, Present, and Future. Cell.

[83] Banikazemi Z., Heidar Z., Rezaee A., Taghavi S.P., Zadeh Modarres S., Asemi Z., Goleij P., Jahed F., Mazaheri E., Taghizadeh M. (2023). Long Non-Coding RNAs and Female Infertility: What Do We Know?. Pathol. Res. Pract..

[84] Xiang S., Yan W., Ren X., Feng J., Zu X. (2024). Role of Ferroptosis and Ferroptosis-Related Long Non’coding RNA in Breast Cancer. Cell. Mol. Biol. Lett..

[85] Yang G., Pan Q., Lu Y., Zhu J., Gou X. (2023). miR-29a-5p Modulates Ferroptosis by Targeting Ferritin Heavy Chain FTH1 in Prostate Cancer. Biochem. Biophys. Res. Commun..

[86] Zhou S.-M., Shi Y., Li J.-Y., Wang N., Zeng Y., Chen H.-Q., Tan Y.-P., Deng S.-W., Liu Q.-Q., Huang X.-Q. (2025). Bisphenol F Induces Spermatogenic Cell Ferroptosis via FTO-Mediated m6A Regulation of FTH1. Free Radic. Biol. Med..

[87] Biamonte F., Battaglia A.M., Zolea F., Oliveira D.M., Aversa I., Santamaria G., Giovannone E.D., Rocco G., Viglietto G., Costanzo F. (2018). Ferritin Heavy Subunit Enhances Apoptosis of Non-Small Cell Lung Cancer Cells through Modulation of miR-125b/P53 Axis. Cell Death Dis..

