# Supplementary material for: Ferritin Heavy Chain 1 Sustains Steroid Hormone Synthesis in Sheep Granulosa Cells by Suppressing Erastin-Induced Ferroptosis
Source: Biomolecules. 2026 Oct 16;16(10):1507. doi: 10.3390/biom16101507 (PMC13702417; doi:10.3390/biom16101507)
Supplement: Supplementary file 1 [file biomolecules-16-01507-s001.zip › Supplementary information.pdf]

Table S1 The CDS region sequence of *FTH1*

**Name**	**Sequences (5′-3′)**
FTH1	ATGACGACCGCATCCCCCTCGCAGGTGCGCCAGAACTACCACCAGGA<br>CTCGGAGGCCGCCATCAACCGCCAGATCAACCTGGAGCTCTACGCCT<br>CCTATGTCTACCTGTCCATGTCGTACTATTTTGACCGTGATGATGTGGC<br>TTTGAAGAACTTTGCCAAATACTTTCTTCACCAATCTCATGAGGAGAG<br>GGAACATGCTGAGAGACTGATGAAGCTGCAGAACCAGCGAGGCGGC<br>CGAATCTTCCTTCAGGATATCAAGAAACCAGACCGTGATGACTGGGA<br>GAATGGGCTGAATGCAATGGAATGTGCGCTGTGCTTGGAAAGAAGTG<br>TGAATCAGTCGCTACTGGAACTGCACAAACTGGCCACTGAAAAAAAT<br>GATCCCCATCTGTGTGATTTCATTGAGACTCATTACCTGAATGAGCAG<br>GTGGAAGCCATCAAAGAATTGGGTGACCACATAACCAACCTGCGCAA<br>GATGGGGGCCCCTGGATCGGGCATGGCAGAGTACCTCTTTGACAAGC<br>ACACCCTGGGACACAGTGACAGCTAA

Table S2 RT-qPCR reaction system

Components	Volume
2×ChamQ SYBR qPCR Master Mix	5 μL
Primer 1 (10 μM)	0.2 μL
Primer 2 (10 μM)	0.2 μL
cDNA	1 μL
ddH$_2$O	3.6 μL

Table S3 RT-qPCR reaction program

Step	Temperature	Time
1	95 ℃	30 s
2	95 ℃	10 s
3	60 ℃	30 s
4	Melting curve	

Table S4 Detection of amplification efficiency of primers

Gene	Slope	Efficiency (%)	(R^2)	Melt peak	Exon spanning
FTH1	-3.326	99.8%	0.9961	Single	yes
ACSL4	-3.376	97.8%	0.9988	Single	yes
STAR	-3.142	108.1%	0.998	Single	yes
HSD17B1	-3.121	109.1%	0.9989	Single	yes

Table S5 Summary of RNA-seq data quality

Sample	Raw Reads	Raw Bases	Clean Reads	Clean Bases	Error rate(%)	Q20(%)	Q30(%)	GC content(%)
CON1	76418998	11539268698	75304696	11177974317	0.0126	98.73	94.91	53.03
CON2	73001718	11023259418	71988802	10720193699	0.0126	98.74	94.97	52.98
CON3	68707696	10374862096	67871692	10131539459	0.0125	98.81	95.12	51.64
Oe_FTH1_1	66936110	10107352610	66176686	9862332052	0.0124	98.84	95.27	51.54
Oe_FTH1_2	75865814	11455737914	74919336	11156739101	0.0127	98.71	94.65	52.04
Oe_FTH1_3	70300204	10615330804	69287404	10275171120	0.0126	98.74	95.01	53.22

Table S6 Summary of read mapping statistics

Sample	Total reads	Total mapped	Multiple mapped	Unique mapped
CON1	75304696	70999880(94.28%)	4117592(5.47%)	66882288(88.82%)
CON3	67871692	64446913(94.95%)	3371929(4.97%)	61074984(89.99%)
CON2	71988802	68109290(94.61%)	3998384(5.55%)	64110906(89.06%)
Oe_FTH1_3	69287404	65205383(94.11%)	3577785(5.16%)	61627598(88.94%)
Oe_FTH1_2	74919336	70603846(94.24%)	3850111(5.14%)	66753735(89.1%)
Oe_FTH1_1	66176686	62593824(94.59%)	3190542(4.82%)	59403282(89.76%)

Table S7 The number of detected genes

RNA Type	Known	Novel	Total
mRNA	48047	10770	58817
lncRNA	8305	1029	9344

S1. Ovarian follicle identification

Follicles with a diameter of 3–4.5 mm were selected for morphological assessment, which was performed based on three criteria: follicular color, vascular abundance, and transparency. RT-qPCR was used to examine the expression of apoptosis-related genes in granulosa cells (GCs) isolated from healthy and atretic follicles. The results showed that the expression levels of *BAX*, *CASP3*, and *CASP9* in the GCs of atretic follicles (AF) were significantly higher than those in healthy follicles, indicating an elevated level of apoptosis and further supporting the accuracy of the follicle morphological classification.

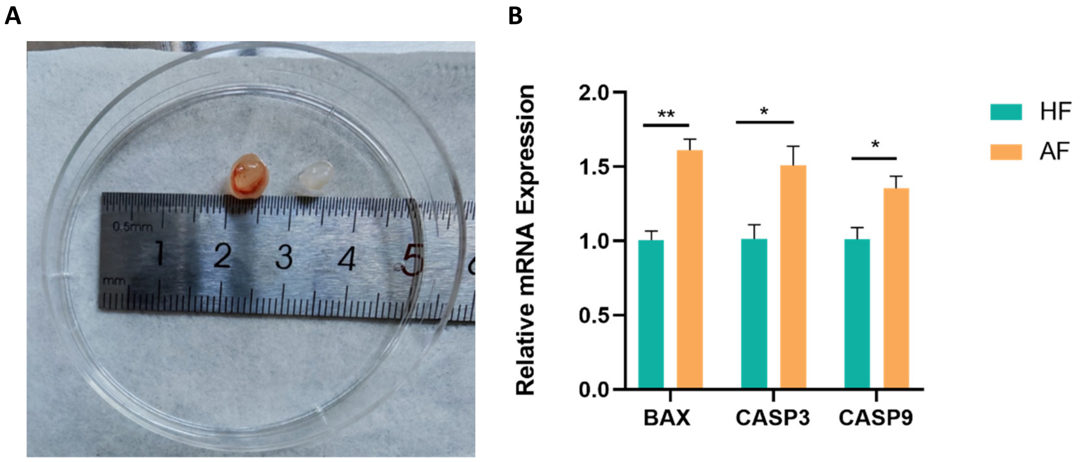

Figure S1. Morphological and biological identification of healthy follicles and atretic follicles.

A: Healthy follicle (HF, left) was translucent with a pale pink or yellow surface and evenly distributed thecal vessels; atretic follicles (AF) feature a grayish-white appearance, flocculent contents, dark wall patches, sparse vasculature and turbid follicular fluid. B: RT-qPCR was used to detect the relative expression levels of *BAX*, *CASP3* and *CASP9* in GCs separately isolated from HF (n = 4 GC samples) and AF (n = 4 GC samples), all 3–4.5 mm in diameter. GCs from 8–12 follicles of the corresponding follicle category were pooled to constitute each sample, with each pooled sample representing an independent biological replicate.

S2. Primary isolation, culture and identification of granulosa cells

Through microscopic observation of cell morphology, it was found that the primary cells cultured in this study were slender spindle-shaped cells, which were consistent with the morphological characteristics of granulosa cells under the microscope (Figure S2A). At the same time, the positive immunofluorescence results of the marker protein FSHR of granulosa cells (Figure S2B) confirmed that the primary ovarian granulosa cells of Hu sheep were isolated and cultured in this study. In addition, sheep preadipocytes were used as a negative control for the immunofluorescence staining of FSHR in granulosa cells (Figure S2C), further confirming that the purity of the isolated granulosa cells was sufficient to support subsequent experiments.

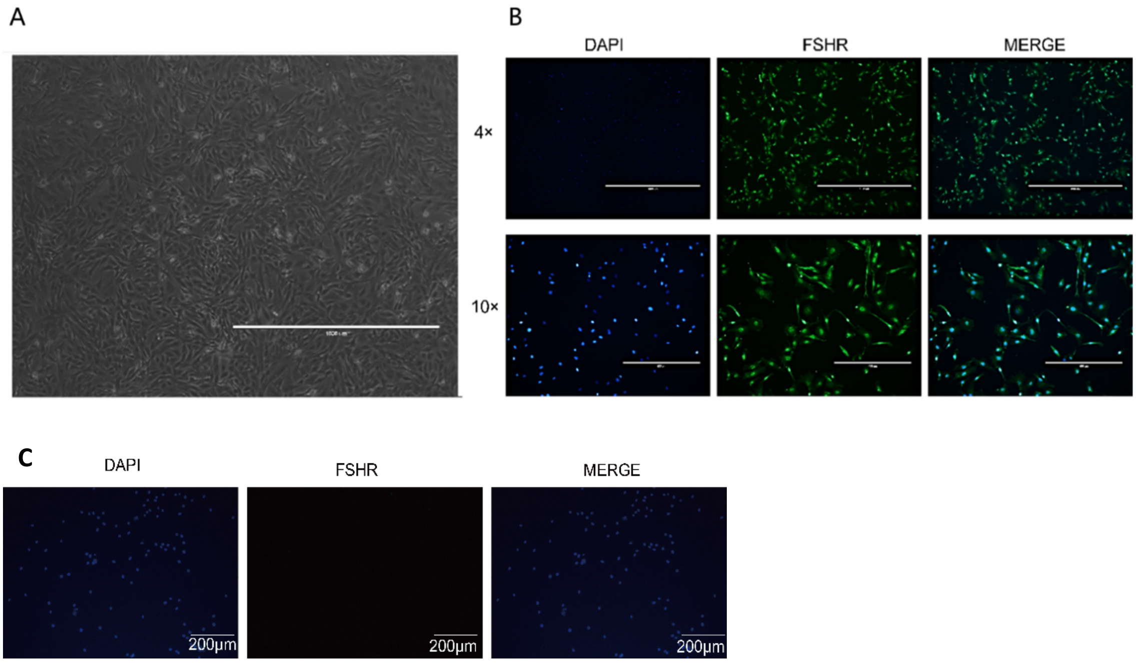

Figure S2. Isolation and identification of primary granulosa cells from Hu sheep ovaries.

A: Microscopic observation of cell morphology, scale bar: 1000μm; B: Immunofluorescent identification of granulosa cells, scale bar: 4×, 1000μm; 10×, 400μm. Blue, DAPI; Green, FSHR; MERGE, composite. C: Sheep preadipocytes were used as a negative control for FSHR

immunofluorescence in granulosa cells. scale bar:10×, 200μm. Blue, DAPI; Green, FSHR; MERGE, composite.

S3. Identification and classification of lncRNAs

Known lncRNAs were identified by comparing the assembled transcripts with available lncRNA annotations from the NONCODE, Ensembl and NCBI databases. For the identification of novel lncRNAs, transcripts assigned the class codes “x”, “i”, “j”, “u” or “o” were initially retained. Candidate transcripts were then required to have a length of at least 200 nt, contain at least two exons and possess an open reading frame no longer than 300 bp. Their protein-coding potential was evaluated using Coding Potential Calculator 2 (CPC2), Coding-Non-Coding Index (CNCI), Coding Potential Assessment Tool (CPAT) and the Pfam protein-domain database. Transcripts with a CPC2 score <0.5, a CNCI score <0, a CPAT score <0.5 and no detectable Pfam protein-domain annotation were considered non-coding. Only transcripts that satisfied the non-coding criteria of all four approaches were retained as novel lncRNA candidates. Based on their genomic positions relative to protein-coding genes, the identified lncRNAs were further classified as sense-exon-overlapping, intergenic, antisense, sense-intron-overlapping or bidirectional. Bidirectional lncRNAs were defined as transcripts located within 1 kb upstream of a protein-coding gene and transcribed in the opposite direction.

S4 Construction of the candidate lncRNA–mRNA trans-regulatory network

The normalized expression profiles of differentially expressed lncRNAs and mRNAs across the six RNA-seq samples were used for trans-association analysis. For each lncRNA–mRNA pair, Pearson's correlation coefficient was calculated. Statistical significance was assessed using a two-sided correlation test. LncRNA–mRNA pairs with $|r| \geqslant 0.6$ and a nomina P <0.05were retained as candidate trans-associated pairs. The retained lncRNAs and mRNAs were represented as network nodes, and significant positive or negative correlations were represented as edges. Because this network was derived from expression correlations, the identified pairs were interpreted as candidate co-expression associations rather than experimentally confirmed regulatory interactions.

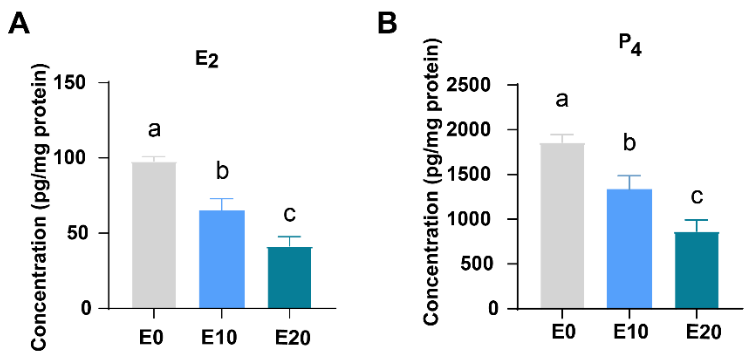

Figure S3. Erastin reduces estradiol and progesterone concentrations in sheep granulosa-cell cultures.

GCs were treated with 0, 10 or 20 µM erastin for 24 h. A: E_2 concentration. B: P_4 concentration. Data are mean ± SEM from three independent biological replicates. Groups with different lowercase letters differ at $P < 0.05$ by one-way ANOVA followed by Fisher's LSD test.

S5 Detection of *FTH1* overexpression efficiency

In order to further study the effect of *FTH1* gene overexpression on GC ferroptosis, the amount of Lipo3000 (0.75 μL and 1 μL) and transfection time (24 h and 48 h) were first selected. As shown by the relative expression of *FTH1* in 24-well plates, In the 24-well plate, 1 μL Lipo3000 was used to transfect the *FTH1* overexpression plasmid for 24 h, which significantly increased the relative expression of *FTH1* ($P < 0.01$) and achieved the effect of overexpressing *FTH1* (Figure S4). Ultimately, we used 1.0 μL of Lipofectamine 3000 for 24 hours as the final transfection system.

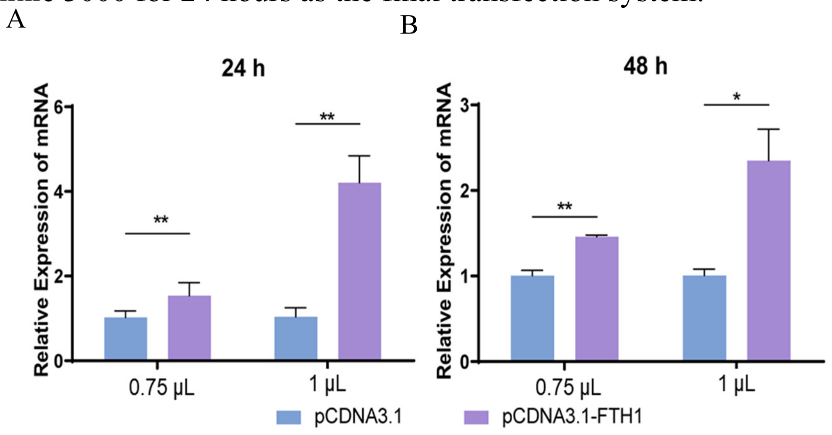

Figure S4. *FTH1* transfection conditions selection.
A-B Relative *FTH1* mRNA after transfection with 0.75 or 1.0 μL Lipofectamine 3000 for 24 or 48 h. Data are mean ± SEM from three independent biological replicates. *$P < 0.05$; **$P < 0.01$ versus the matched control.

S6. Detection of *FTH1* interference RNA efficiency

In order to screen the appropriate *FTH1* interfering RNA, the expression of *FTH1* after transfection of the three candidate siRNAs was detected, and it was found that 24 h after transfection, all the three siRNAs could significantly reduce the expression of *FTH1* ($P < 0.05$), among which si1 had the best effect (Figure S4). Therefore, si1 was ultimately selected for the subsequent experiments, and was named si-*FTH1*.

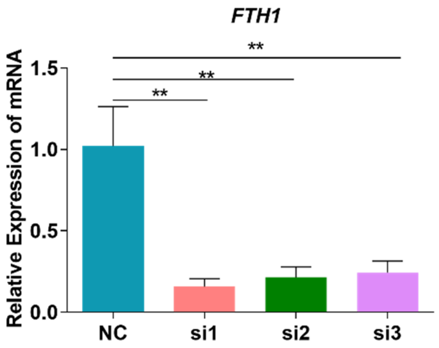

Figure S5. Selection of *FTH1*-targeting siRNA in sheep granulosa cells. Relative *FTH1* expression was measured 24 h after transfection with candidate siRNAs and the negative-control siRNA. Data are mean ± SEM from three independent biological replicates. *$P < 0.05$; **$P < 0.01$ versus the matched control.

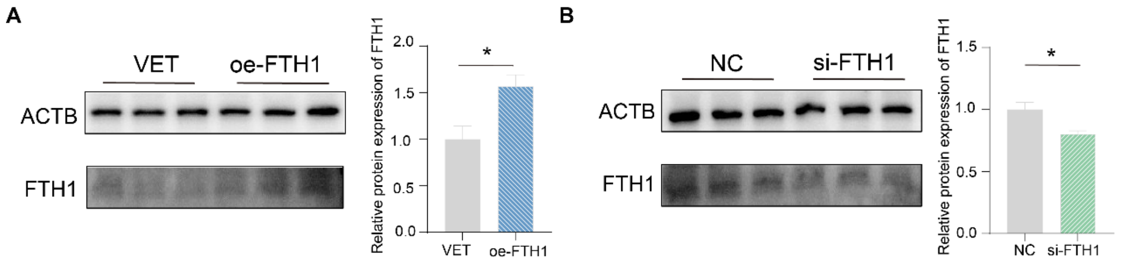

Figure S6. protein-level validation of *FTH1* overexpression and interference

A-B: Representative *FTH1* western blots after oe-*FTH1* and si-*FTH1* transfection 24h. Densitometric quantification normalized to β-actin. Data are mean ± SEM from three independent biological replicates. *$P < 0.05$ versus the matched control.

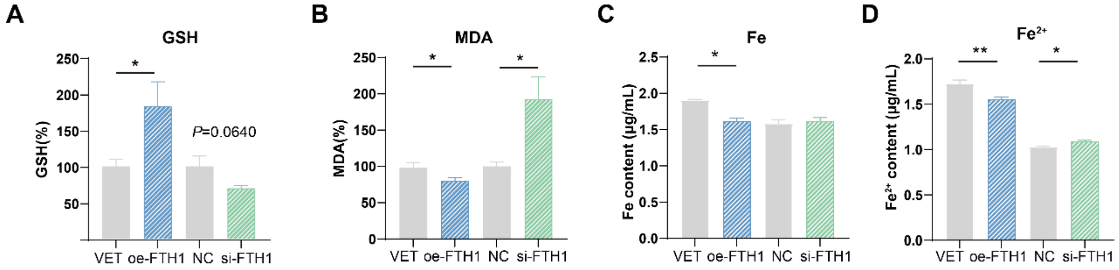

Figure S7. Effects of *FTH1* on ferroptosis-related biochemical indicators without erastin.

GCs were transfected with empty vector or oe-*FTH1*, or with negative-control siRNA or si-*FTH1*, without erastin treatment. A: Relative GSH content. B: Relative MDA content. C: Total iron after *FTH1* overexpression and interference. D: Fe^{2+} after *FTH1* overexpression and interference. Data are mean ± SEM from three independent biological replicates. *$P < 0.05$; the exact P value is shown where applicable.

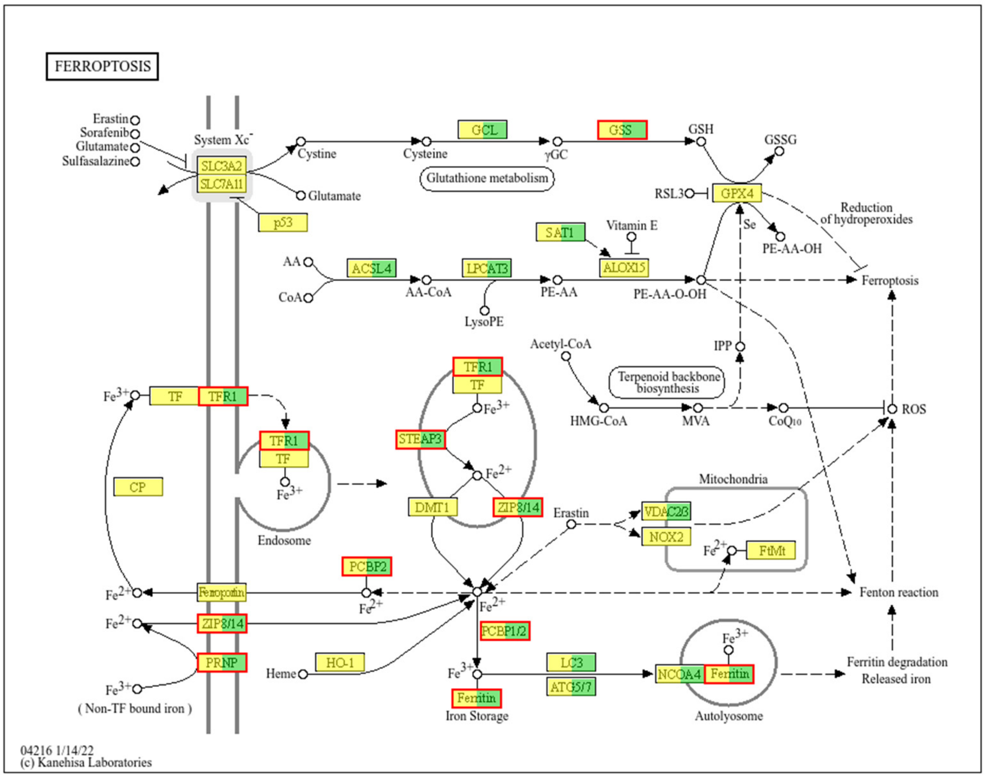

Figure S8. Expression changes in ferroptosis-pathway genes after *FTH1* overexpression under erastin stress.

Red borders indicate genes with increased expression, and blue borders indicate genes with decreased expression in oe-*FTH1* cells relative to vector controls under 10 µM erastin.
