# Supplementary material for: Ferritin Heavy Chain 1 Sustains Steroid Hormone Synthesis in Sheep Granulosa Cells by Suppressing Erastin-Induced Ferroptosis
Source: Biomolecules. 2026 Oct 16;16(10):1507. doi: 10.3390/biom16101507 (PMC13702417; doi:10.3390/biom16101507)

Protein Marker was SB-26616 from Share-Bio. Given the use of a polyclonal antibody for CYP11A1 immunoblotting, additional bands presumably result from cross-reactions with off-target proteins. The band near 60 kDa, consistent with its anticipated molecular mass, was subjected to quantitative analysis; other bands were interpreted as possible non-specific immunoreactive products.

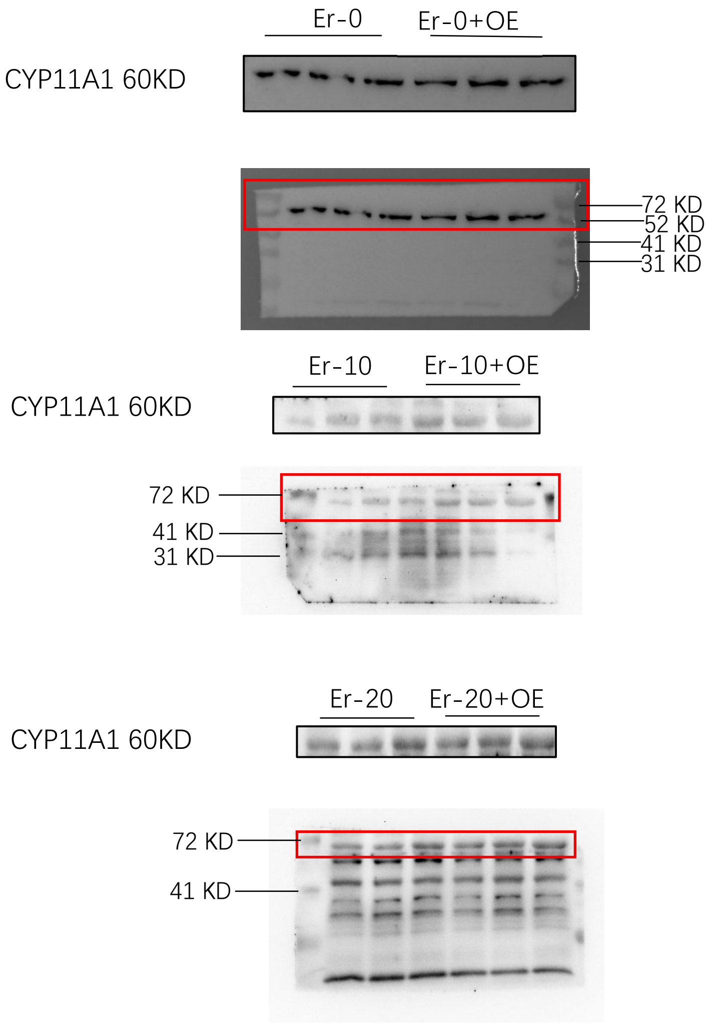

CYP11A1 60KD

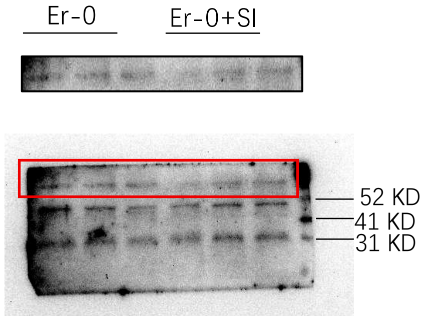

CYP11A1 60KD

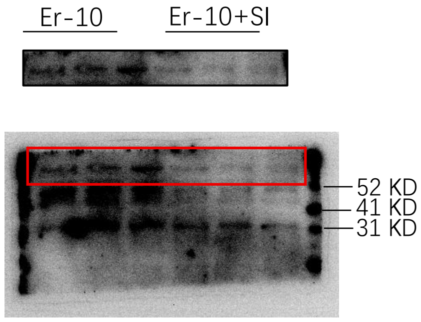

CYP11A1 60KD

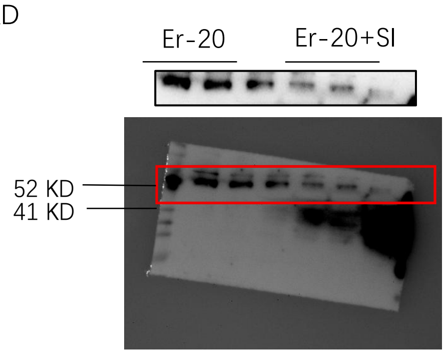

Supplement: Supplementary file 1 [file biomolecules-16-01507-s001.zip › Original Images for Blots.zip!/√CYP11A1.pdf]
