# Supplementary material for: Ferritin Heavy Chain 1 Sustains Steroid Hormone Synthesis in Sheep Granulosa Cells by Suppressing Erastin-Induced Ferroptosis
Source: Biomolecules. 2026 Oct 16;16(10):1507. doi: 10.3390/biom16101507 (PMC13702417; doi:10.3390/biom16101507)

β-actin 42 KD:

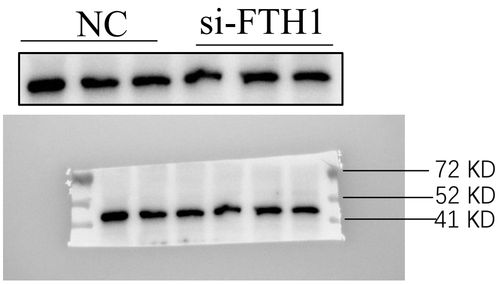

FTH1 21KD: As a polyclonal antibody was used to detect FTH1, the additional bands were likely caused by cross-reactivity with other proteins. The band at approximately 21 kDa was considered to represent FTH1 because it increased after FTH1 overexpression and decreased after FTH1 knockdown. Therefore, only this band was included in the quantitative analysis.

FTH1 21 KD

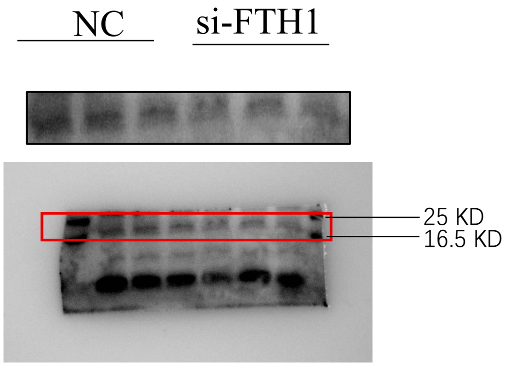

β-actin 42 KD

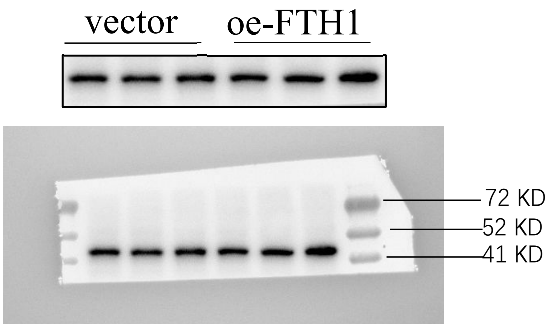

FTH1 21 KD

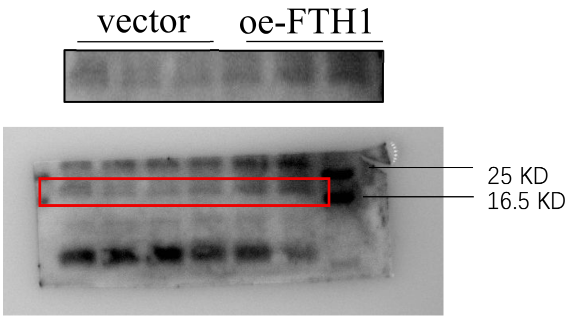

Supplement: Supplementary file 1 [file biomolecules-16-01507-s001.zip › Original Images for Blots.zip!/√Figure S4 FTH1 & β-actin.pdf]
