# Supplementary material for: Ferritin Heavy Chain 1 Sustains Steroid Hormone Synthesis in Sheep Granulosa Cells by Suppressing Erastin-Induced Ferroptosis
Source: Biomolecules. 2026 Oct 16;16(10):1507. doi: 10.3390/biom16101507 (PMC13702417; doi:10.3390/biom16101507)
Supplement: Supplementary file 1 [file biomolecules-16-01507-s001.zip › Original Images for Blots.zip!/√GPX4 & Casp3.pdf]

Er-0 Er-0+OE

GPX4 22KD

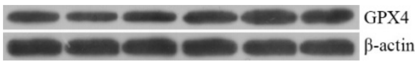

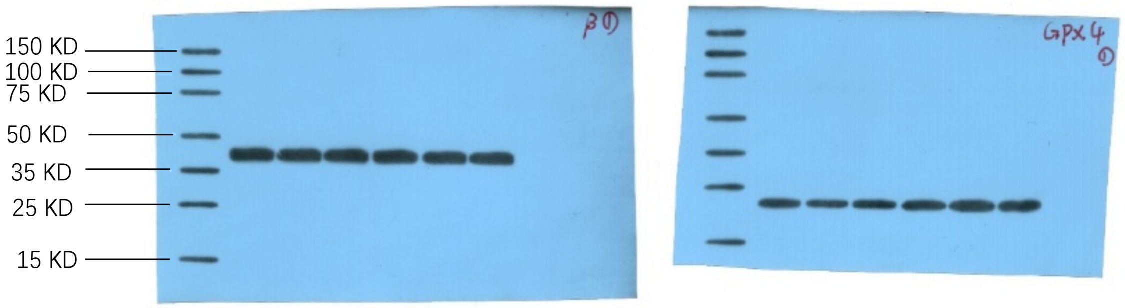

GPX4 22KD

Er-10 Er-10+OE

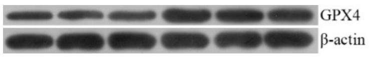

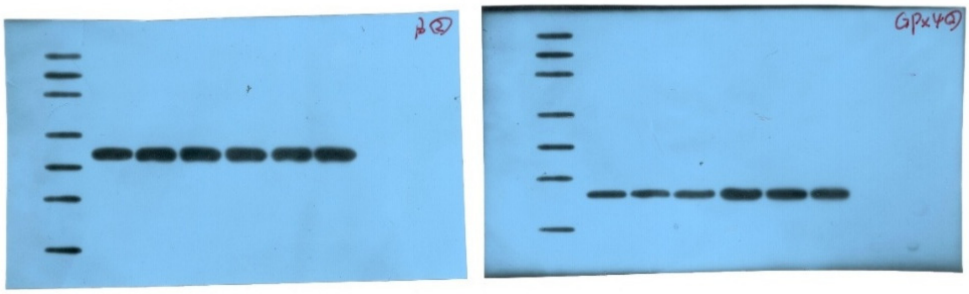

Er-20 Er-20+OE

GPX4 22KD

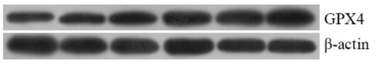

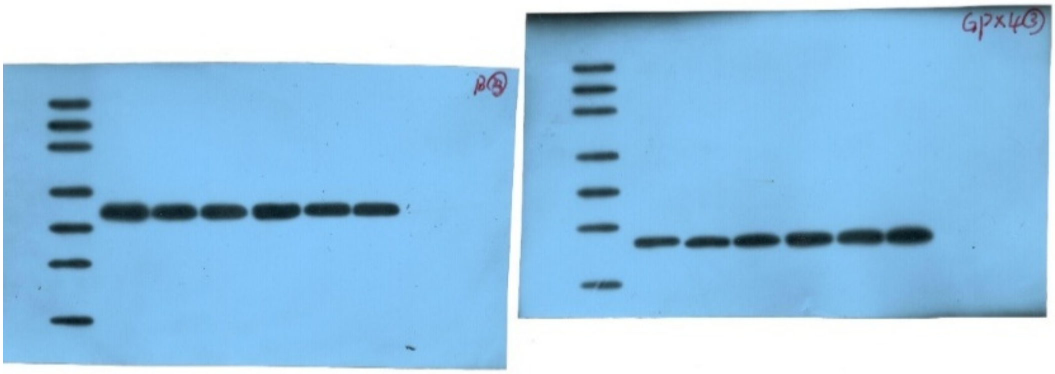

GPX4 22KD

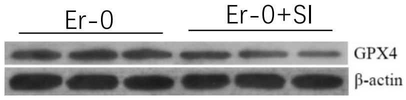

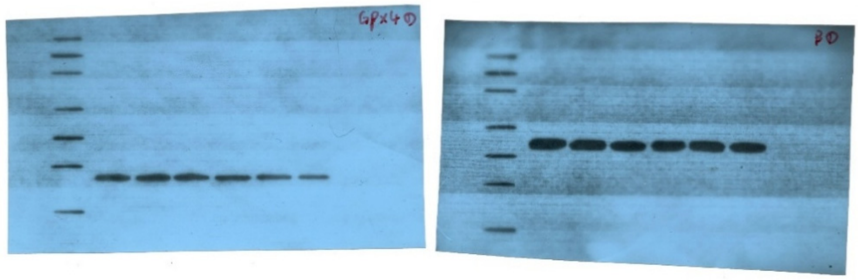

GPX4 22KD

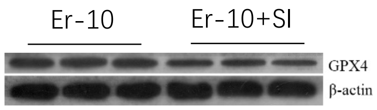

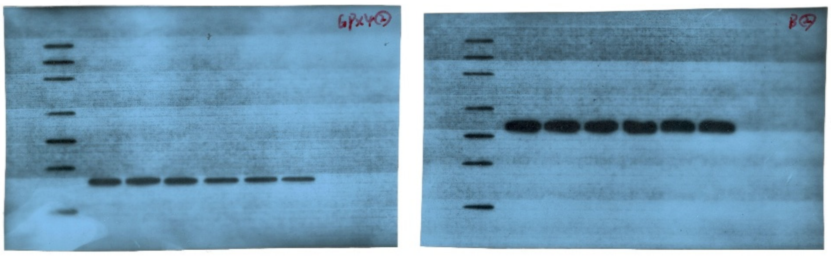

GPX4 22KD

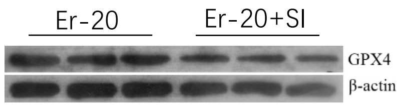

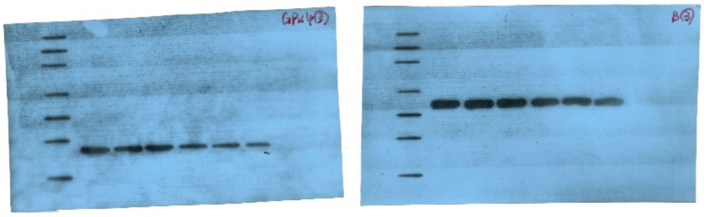

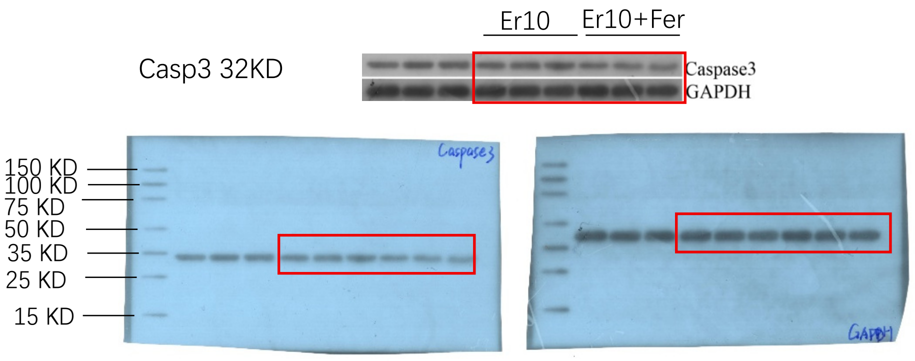
Casp3 32KD
Er10
Er10+Fer
Caspase3
GAPDH
150 KD
100 KD
75 KD
50 KD
35 KD
25 KD
15 KD
