# Supplementary material for: Ferritin Heavy Chain 1 Sustains Steroid Hormone Synthesis in Sheep Granulosa Cells by Suppressing Erastin-Induced Ferroptosis
Source: Biomolecules. 2026 Oct 16;16(10):1507. doi: 10.3390/biom16101507 (PMC13702417; doi:10.3390/biom16101507)

Protein Marker was SB-26616 from Share-Bio. As a polyclonal antibody was used to detect STAR, the additional bands were likely caused by cross-reactivity with other proteins. The band at approximately 25 kDa, which was closest to the expected molecular-weight range of STAR, was used for quantitative analysis. The other bands were considered potentially nonspecific immunoreactive signals.

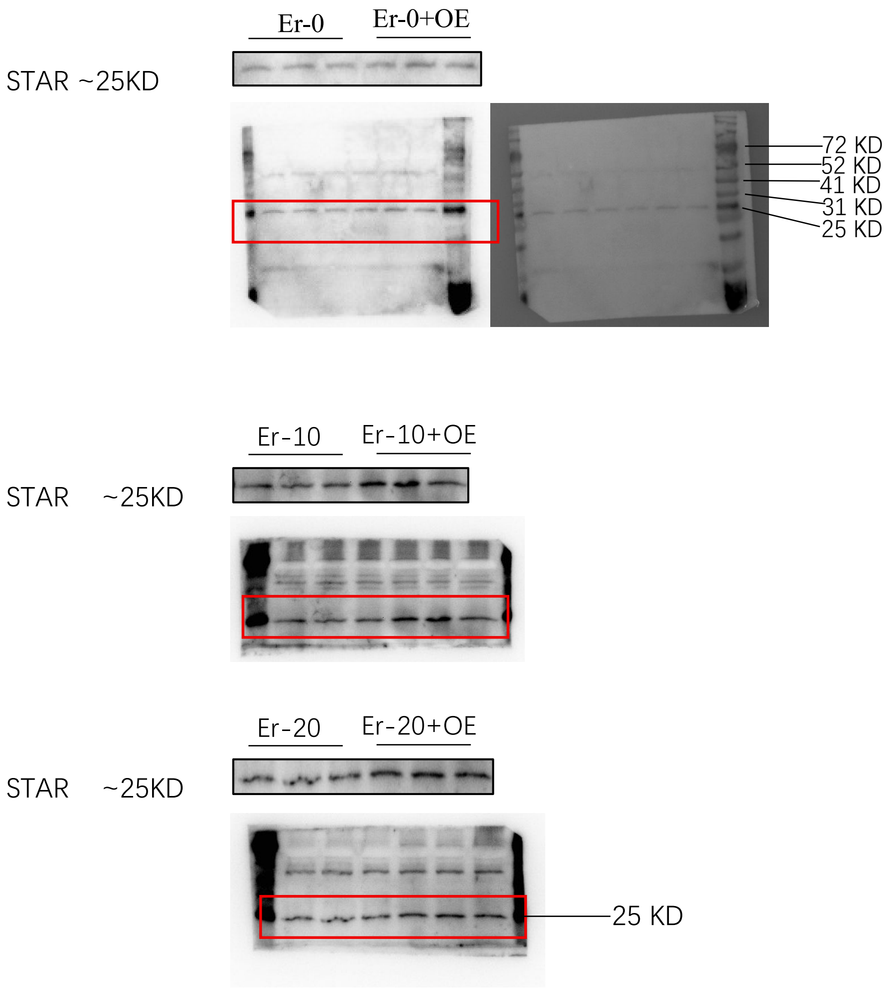

STAR ~25KD

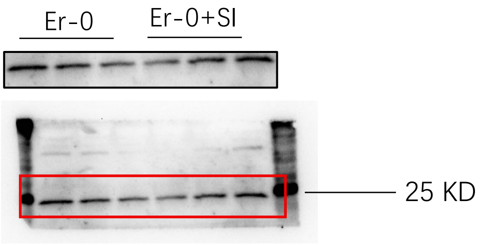

STAR ~·25KD

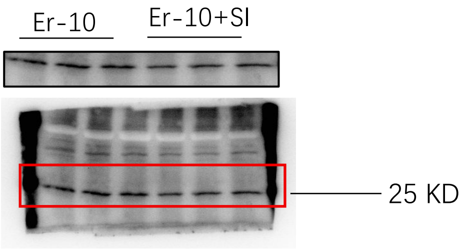

STAR ~25KD

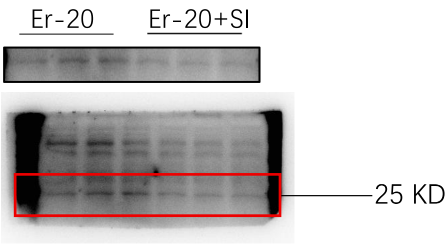

Supplement: Supplementary file 1 [file biomolecules-16-01507-s001.zip › Original Images for Blots.zip!/√STAR.pdf]
