# Supplementary material for: Ferritin Heavy Chain 1 Sustains Steroid Hormone Synthesis in Sheep Granulosa Cells by Suppressing Erastin-Induced Ferroptosis
Source: Biomolecules. 2026 Oct 16;16(10):1507. doi: 10.3390/biom16101507 (PMC13702417; doi:10.3390/biom16101507)

ACTB 42KD

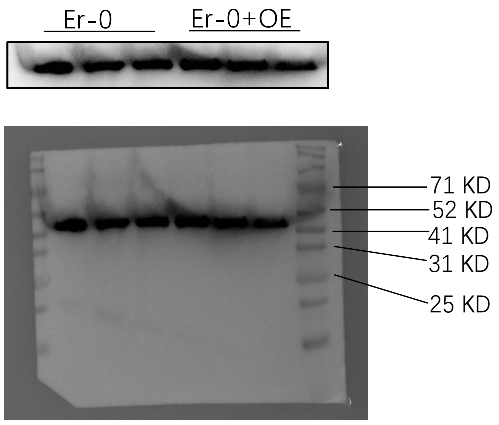

ACTB 42KD

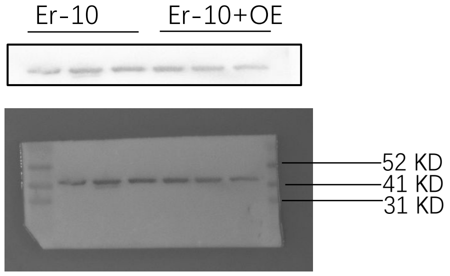

ACTB 42KD

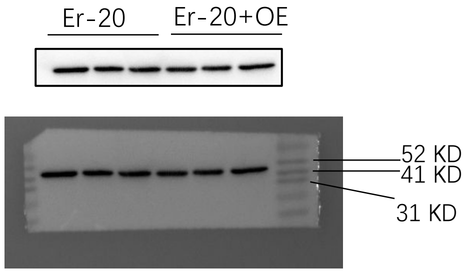

ACTB 42KD

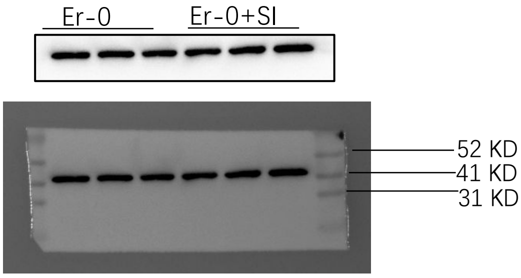

ACTB 42KD

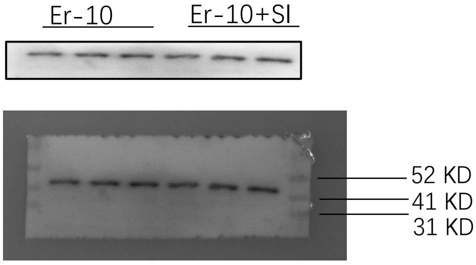

ACTB 42KD

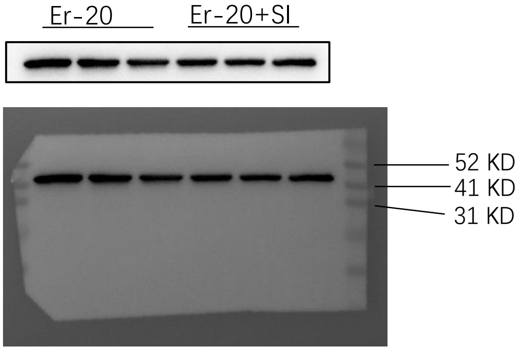

Supplement: Supplementary file 1 [file biomolecules-16-01507-s001.zip › Original Images for Blots.zip!/√β-actin.pdf]
